# Probing the Dynamics of Yersinia Adhesin A (YadA) in Outer Membranes Hints at Requirements for β-Barrel Membrane Insertion

**DOI:** 10.1021/jacs.4c17726

**Published:** 2025-02-27

**Authors:** Jayasubba Reddy Yarava, Marcella Orwick-Rydmark, David Ryoo, Albert Hofstetter, James C. Gumbart, Michael Habeck, Barth-Jan van Rossum, Dirk Linke, Hartmut Oschkinat

**Affiliations:** † Leibniz-Forschungsinstitut für Molekulare Pharmakologie, Robert-Rössle-Straße 10, 13125 Berlin, Germany; ‡ Freie Universität Berlin, Takustraße 3, 14195 Berlin, Germany; § Department of Biosciences, University of Oslo, P.O.Box 1066 Blindern, 0316 Oslo, Norway; ∥ Interdisciplinary Bioengineering Graduate Program, Georgia Institute of Technology, Atlanta, Georgia 30332, United States; ⊥ Department of Chemistry and Applied Biosciences, ETH Zurich, Vladimir-Prelog-Weg 2, 8093 Zurich, Switzerland; # School of Physics, Georgia Institute of Technology, Atlanta, Georgia 30332, United States; ∇ Microscopic Image Analysis Group, Jena University Hospital, Am Klinikum 1, 07747, Jena, Germany

## Abstract

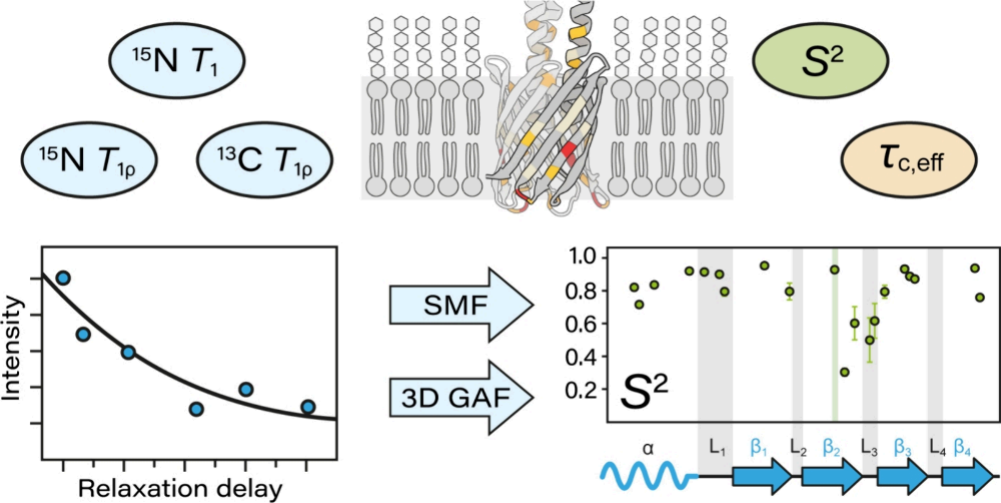

The vast majority of cells are protected and functionalized by a dense surface layer of glycans, proteoglycans, and glycolipids. This surface represents an underexplored space in structural biology that is exceedingly challenging to recreate in vitro. Here, we investigate β-barrel protein dynamics within an asymmetric outer membrane environment, with the trimeric autotransporter Yersinia adhesin A (YadA) as an example. Magic-angle spinning NMR relaxation data and a model-free approach reveal increased mobility in the second half of strand β2 after the conserved G72, which is responsible for membrane insertion and autotransport, and in the subsequent loop toward β3. In contrast, the protomer–protomer interaction sites (β1_*i*_–β4_*i*–1_) are rigid. Intriguingly, the mobility in the β-strand section following G72 is substantially elevated in the outer membrane and less so in the detergent environment of microcrystals. A possible source is revealed by molecular dynamics simulations that show the formation of a salt bridge involving E79 and R76 in competition with a dynamic interplay of calcium binding by E79 and the phosphate groups of the lipids. An estimation of overall barrel motion in the outer membrane and detergent-containing crystals yields values of around 41 ns for both. The global motion of YadA in the outer membrane has a stronger rotational component orthogonal to the symmetry axis of the trimeric porin than in the detergent-containing crystal. In summary, our investigation shows that the mobility in the second half of β2 and the loop to β3 required for membrane insertion and autotransport is maintained in the final folded form of YadA.

## Introduction

**Figure 1:**
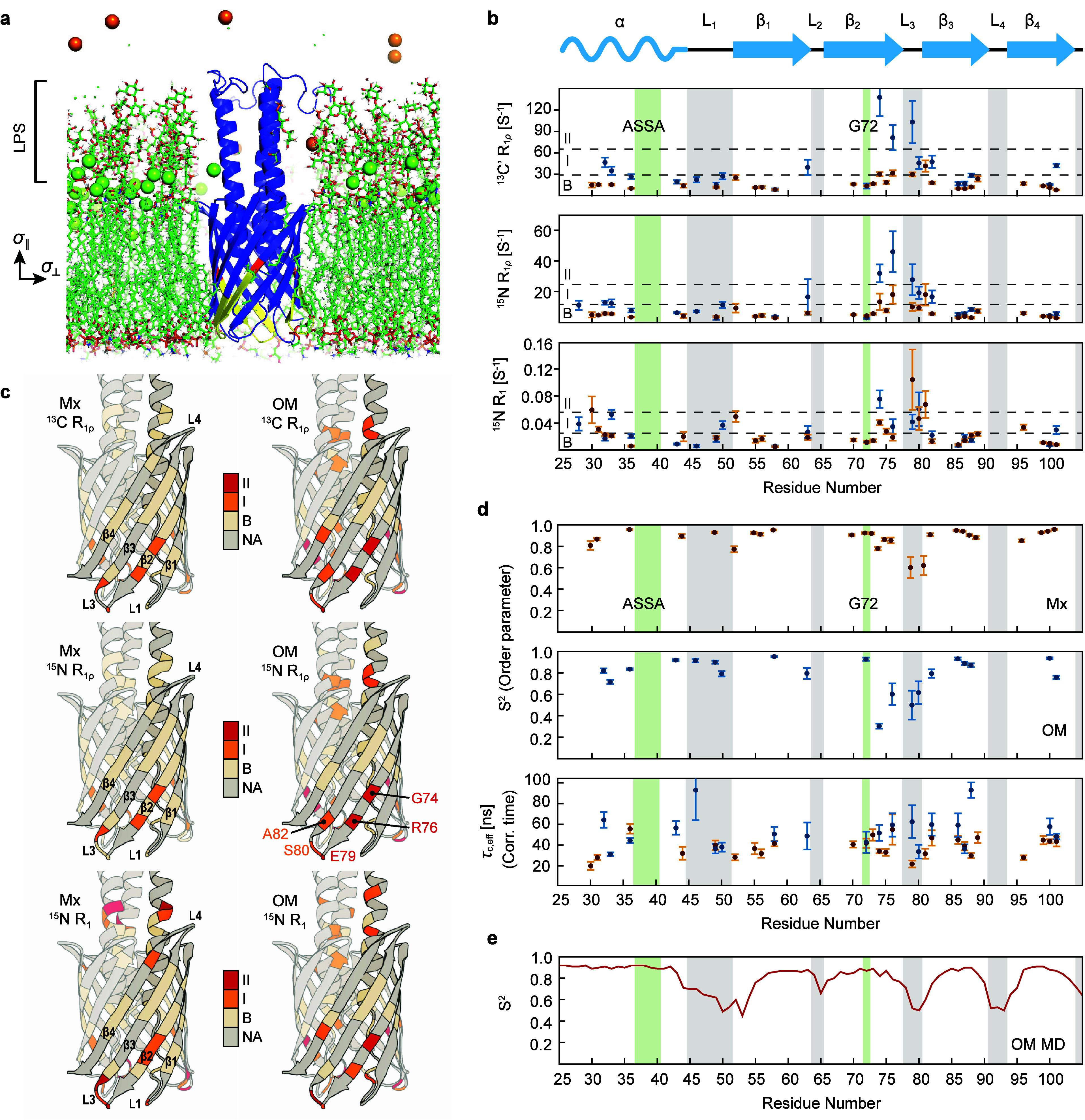
Dynamics properties of YadAM in OMs and the microcrystalline environment. (a) YadA anchor domain (YadAM) in an asymmetric bacterial OM environment. The outer leaflet contains LPS, whereas the inner leaflet contains mostly phospholipids. Membrane axes as used in this study are shown on the left. G72 is colored red, and the second half of β2 is shown in yellow. Green spheres are calcium ions, and orange spheres are potassium ions. (b) Experimentally determined ^15^N *R*_1_ and ^15^N as well as ^13^C′ *R*_1ρ_ relaxation rates of YadAM-Mx (orange) and OM (blue). The gray bars represent loop regions, and green bars represent the residues that are involved in autotransporter (ASSA) and membrane insertion (G72) mechanisms. The black dashed lines define the classes employed as the color code in (c). The three classes of relaxation times are defined as fast (II, red), medium (I, orange), and base (B, ivory). The ^15^N R_1_ rates were categorized as 0–0.025 s^–1^ (B), 0.025–0.056 s^–1^ (I), and >0.056 s^–1^ (II). The ^15^N R_1ρ_ rates were categorized as 0–12 s^–1^ (B), 12–25 s^–1^ (I), and >25 s^–1^ (II). The ^13^C *R*_1ρ_ rates were categorized as 0–29 s^–1^ (B), 29–66 s^–1^ (I), and >66 s^–1^ (II). (c) Visualization of relaxation times sorted into three classes; see the dashed lines in (b). All analyzed residues appear in red, orange, or ivory. Nonanalyzed residues are in gray. One protomer of the trimeric barrel is shown with a slightly darker gray shade. (d) Interpretation of the ^15^N *R*_1_ and *R*_1ρ_ relaxation rates with the SMF formalism. Order parameters for YadAM-Mx and YadAM-OM are shown in the top and center panels, respectively. Correlation times are shown in the lower panel, with values depicted in blue for YadAM-OM and in orange for YadAM-Mx. (e) MD-derived order parameters of YadAM-OM at the NH peptide plane vectors were obtained from 2 μs MD simulations in a realistic OM model. The values represent the average of 3 replicas.

The outer membrane (OM) of Gram-negative bacteria consists of lipopolysaccharides (LPS) in the outer leaflet, while the inner leaflet is mostly composed of phospholipids.^1^ This asymmetry extends into the lipid sections, with unsaturated fatty acids present only in the inner leaflet and lipids with stronger varying chain lengths in the outer one.^2^ As a major obstacle to obtaining the most realistic picture of membrane protein structure and dynamics, it is notoriously difficult to recreate such asymmetry in vitro. Yet, regarding understanding the insertion of proteins into the OM, for example, the influence of the lipid bilayer has been highlighted^3^ such that experimental protein dynamics investigations require an in situ-like situation. Furthermore, within the LPS carbohydrate layer, charged moieties such as sulfate and phosphate groups determine the local pH and coordinate divalent cations. Dense hydrogen-bonding networks contribute to the rigidity and gel-like properties, potentially featuring links to proteinaceous interaction partners. The LPS carbohydrate layer has a thickness that exceeds that of the actual membrane bilayer. This thickness strongly depends on the repeat number of O-antigen chains, which vary in length and composition depending on the bacterial species or strain. The otherwise rather impenetrable carbohydrate layer is disturbed above the positions of the membrane-integrated proteins (Figure 1a). They create wide open spaces resembling funnels through the LPS layer, where the loops of an outer membrane protein (Omp) that are observed as flexible in artificial membrane bilayers may become restricted in motion. Such a restriction possibly leads to structural ordering for entropic reasons or due to the formation of hydrogen bonds with the oligosaccharide environment and may even affect the dynamics of protein segments within the lipid sections of the bilayer. It is to be expected that even small influences change the loop structures of the Omps, as reflected in the varying structural biology results. X-ray structures show such loops to be frequently well ordered, often stabilized by crystal contacts, whereas NMR tends to detect considerable flexibility for the same moieties depending on the membrane substitute employed in vitro. For example, outer membrane protein G (OmpG) exhibits highly flexible extracellular loops 6 and 7 in detergent solutions, intermediate flexibility in model lipid bilayers, and a well-ordered structure in a three-dimensional crystalline environment.^4,5^ Given the variability of loop structures in Omps and the different lipid compositions of both leaflets, investigations of the structural influence of the asymmetric OM on their dynamics are important, especially as membrane biogenesis is linked to the dynamics properties of the involved proteins and the lipid environment. These include outer membrane insertion of proteins by the β-barrel assembly machinery (BAM),^6^ autotransport of the extracellular domains of adhesins,^7^ and the opening/closing of porins.^8^

The transmembrane units of the trimeric autotransporter YadA serve as interesting examples of β-barrel proteins.^9^ YadA is a virulence factor found in the OM of the pathogens *Yersinia enterocolitica* and *Yersinia pseudotuberculosis*.^10^ Three protomers of YadA contribute four C-terminal β-strands per protomer to a formally symmetric 12-stranded β-barrel that is investigated here together with a short fragment of its helical stalk, in the following referred to as YadAM. In full-length YadA, the homotrimeric transmembrane domain anchors the adhesin in the membrane and mediates self-export of the three N-terminal, extended extracellular adhesin domains.^11^ Mutational studies^12,13^ and structural models derived by solid-state NMR (ssNMR) of microcrystalline samples (for YadAM^14^) and by X-ray crystallography (for the YadA homologue Hia from *Haemophilus influenza*(15)) have suggested several conserved residues in the transmembrane domain of trimeric autotransporter adhesins (TAAs) that directly influence either membrane insertion or autotransport function. Efficient membrane insertion and structural stability are linked to a glycine residue in the third-last β-strand that is perfectly conserved across TAAs from different species. Mutations of this glycine residue to larger residues negatively affect the surface display of the adhesin.^12^ Conserved glycine residues play an important role in the membrane insertion and stability of β-barrel proteins, in general, extending beyond the autotransporter family. They form pairs with tyrosine residues in a neighboring β-strand, which are supposed to stabilize folding intermediates as well as the final protein structure. This has been extensively demonstrated for the model protein OmpX, where such an interaction contributes to a folding core,^16^ and where mutations lead to a destabilization of the final structure and to slower folding in detergent solution.^17^ Similar Gly–Tyr pairs, also known as “mortise-tenon motifs”, have been identified in autotransporter proteins and in porins of diverse sizes and strand numbers from small, 8-strand barrels to large porins^18^ with 16 or more strands.^17^ The structural^14,15^ and mutational studies^12,13^ of YadA suggest further that a conserved stretch of small residues, the “ASSA” region, can adopt different conformations during and after autotransport—a flexible loop that initiates the transport process by forming a hairpin and later allowing the rest of the unfolded protein chain to slip through the transmembrane pore.

The measurement and evaluation of ssNMR relaxation parameters, such as the spin–lattice relaxation rate (*R*_1_) and the spin–lattice relaxation rate in the rotating frame (*R*_1ρ_), comprise a valuable technique for probing protein dynamics, providing insights into motions across multiple time scales.^19−38^ In this study, we investigate the backbone dynamics of YadAM in outer membranes (YadAM-OMs) by measuring the ^15^N *R*_1_, ^15^N *R*_1ρ_, and ^13^C′ *R*_1ρ_ relaxation rates under fast magic-angle spinning (MAS) NMR conditions. These rates are analyzed on a residue-by-residue basis using the simple model-free (SMF) approach,^19,20,27,39^ while the overall motion of the integrated β-barrel is investigated on a collective basis using a 3D Gaussian axial fluctuation (3D GAF) model.^21,28,29,40^ In previous studies, we have thoroughly investigated YadAM in a detergent coat via a microcrystalline preparation (YadAM-Mx); hence, conditions and full chemical shift assignments for this kind of sample are available. This gave us the opportunity to include YadAM-Mx in the current study as a system that is well understood and that allows for judging the plausibility of results at a higher signal-to-noise level. There are two features of this sample that make a difference to YadAM-OM: the embedding of YadAM into a detergent coat and the motional restrictions of the crystalline state. Although YadA-Mx has less biological significance than the YadAM-OM sample, we discuss and compare the determined values of both systems at individual points, especially regarding the detergent-covered area of the barrel including loop regions. A potential benefit of this approach lies in the opportunity to bridge to the majority of membrane protein structures deposited in the protein data bank that are mostly solved after detergent solubilization and protein crystallization. With this study, we want to contribute to an ongoing discussion concerning the importance of the native environment—in this case, the asymmetric OM of Gram-negative bacteria—on structure and dynamics.

This work is based on earlier investigations concerning the expression of YadAM directly into the bacterial OM.^41^ In this previous work, samples were obtained that did not yield spectra with sufficient signal-to-noise for a study as presented here, and additional signals were observed that could only be understood as caused by impurities such as other porins (see Supplementary Figure 3 of ref (41)) next to the expected lipid signals. In the context of the work presented here, expression was vastly improved making use of a specialized *E. coli* strain with multiple Omps (OmpA, C, F, and bacteriophage lambda receptor B (LamB)) deleted.^42^ This resulted in more YadAM integrating into the OM relative to other proteins and, together with improved protocols to separate the OMs from the inner membranes after cell lysis, led to a further reduction of background signals in the NMR spectra and an improved signal-to-noise.

## Results

### Resonance Assignment

In the context of the current investigation, relaxation times were measured as relaxation-dependent intensity changes of cross peaks in 2D NCO spectra (Figure S1 and Text S1) using the pulse sequences shown in Figure S2. Experimental parameters are listed in Table S1. The evaluation of these 2D spectra required the sequence-specific assignment of the NCO cross peaks in the 2D correlation of the OM sample. To this end, the ssNMR ^13^C and ^15^N resonance assignments of YadAM in the microcrystalline form were utilized.^14,43^ However, in the previous study, a spectrometer with a field of 9.1 T was used; hence, a transfer of the assignments to the spectra recorded at 21.1 T was also necessary. Therefore, we checked on the positions of the amide nitrogen and C′ resonances in the 2D NCO and NCA correlation spectra of YadAM-Mx to avoid errors due to slight chemical shift differences in comparison to the previous study conducted at 9.1 T. The ^15^N chemical shifts of most residues were identified via three different cross peaks, involving the C′ chemical shift of the residue, of its own Cα, and of the Cα chemical shifts of the preceding residue. The assignments in the NCO spectrum of YadAM-Mx were then transferred to the NCO spectra of YadAM-OM for instances of sufficient proximity or overlap of cross peaks (Figure S1). The cross peaks in the OM spectrum that were used for the evaluation of relaxation times are indicated in Figure S1B.

### Relaxation Rate Measurements

The ^15^N *R*_1_ and *R*_1ρ_ as well as ^13^C′ *R*_1ρ_ relaxation rates were measured with standard pulse sequences (Figure S2). The rates were obtained after fitting the observed intensity changes to simple exponentials (Figure 1b), and values are listed in Table S2. The magnetization decay curves of YadAM-Mx and YadAM-OM are shown in Figures S3 (^15^N *R*_1_), S4 (^15^N *R*_1ρ_) and S5 (^13^C′ *R*_1ρ_). In general, the ^15^N *R*_1_ are sensitive to fast motions in the pico- to nanosecond (ps–ns) time range, whereas ^15^N *R*_1ρ_ and ^13^C’ *R*_1ρ_ are sensitive to slower, nano- to microsecond range (ns−μs) motions. Comparing the determined values for YadAM in the two different environments, we observe similar ^15^N *R*_1_ (Figure 1b, bottom panel) with deviations at the termini and the second half of β2 and in L3. On the other hand, the ^15^N *R*_1ρ_ values are on average higher for YadAM-OM than for YadAM-Mx (Figure 1b, center). The most pronounced local differences are observed for the ^13^C′ *R*_1ρ_ values (Figure 1b, top). There, the helical stem shows increasing values toward the N-terminus for the OM sample and larger ones in the region 72–80 for the OM than for the microcrystalline preparation. Altogether, the relative trends along the primary sequences are similar, except for the helical stem region and the residues beyond G72 and L3, where systematically higher values were obtained for the OM sample than for the crystalline one. This is apparent from the display of the values on the structure of the YadAM trimer (Figure 1c), defining three categories (B for baseline, ivory; I, orange; II, red) according to the horizontal lines drawn in Figure 1b.

In some cases, a counterintuitive relaxation behavior was observed. For example, for both YadAM-Mx and YadAM-OM, residue R76 shows a close-to-average ^15^N *R*_1_ value but relatively high values in the *R*_1ρ_ measurements. A similar behavior is observed for residues F46 and Y63. In some cases, we observed the opposite trend, where the *R*_1_ values are larger than the average and the *R*_1ρ_ values are not elevated such as for residues T30, R31, and S44. This indicates that different types of motion at different time scales can be present. To characterize the amplitudes and correlation times of the underlying motions in more detail, we analyzed the measured relaxation rates via the procedures described next.

### Local Dynamics by the SMF Approach

We employed an SMF approach to analyze the ^15^N *R*_1_ and *R*_1ρ_ as well as ^13^C′ *R*_1ρ_ relaxation rates with respect to residue-specific dynamics (Texts S3 and S4).^26,27^ This approach provides a single amplitude of the isotropic motion and an averaged correlation time per residue (Figure 1d and Table S3). Back-calculation of relaxation times from the obtained values yielded a satisfactory fit for both samples (Text S5, Figures S6, S7, and Table S4). Since the β-barrel is relatively rigid, high-order parameters (*S*^2^ > 0.8) are obtained for three of the four strands, specifically β1, β3, and β4 (Figure 1d, upper panels), with a gradual decrease toward their edges. In the OM data, high-order parameters are found in L1 except for the short transition to β1 (*S*^2^ = 0.8–0.9 for YadAM-OM). This loop comprises four hydrophobic residues and is located inside the barrel, limiting its motional freedom. Surprisingly, the end of β2 from G74 onward and L3 exhibit very low-order parameters (*S*^2^ = 0.3–0.8). Intriguingly, this effect is more pronounced for the OM sample, hinting at a possible biological relevance of the detected flexibility during the membrane insertion process for which G72 is critical. The successive strands β3 and β4 are again relatively rigid, with signs of higher dynamics toward L4 for which we could not extract data. In our previous studies, we observed indications of enhanced mobility in the conserved ASSA region (residues 37–40) based on TALOS analysis and cross-polarization build-up rates.^41,44^ Unfortunately, the cross peaks of these four residues were not sufficiently separated from the others in the NCO correlation experiment, preventing us from analyzing their dynamic properties.

**Figure 2:**
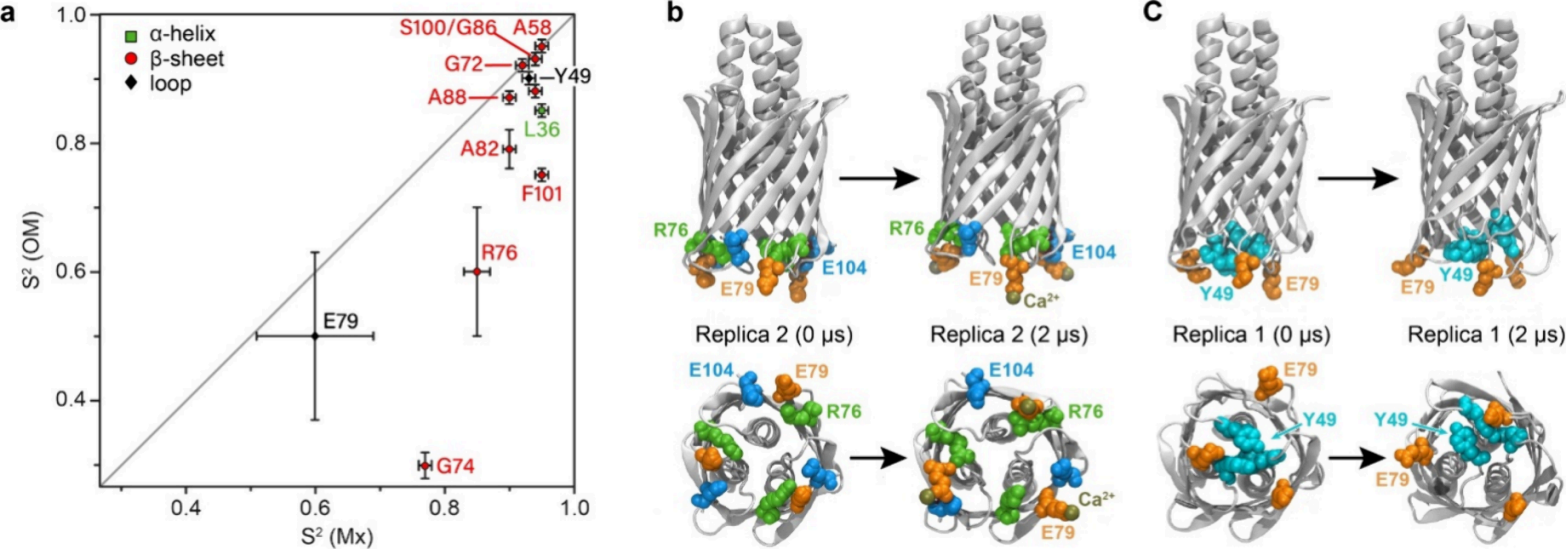
Structural variability at the intracellular face of YadAM. (a) Correlation of OM and Mx *S*^2^ values. (b) Calcium ions bound to a highly conserved region (residues 75–80) (see also Figure S10 for ions bound vs time). (c) Alternative interactions between E79 and Y49.

Several order parameters for residues in the β2/L3 region are lower in the OM environment than in the crystal. The difference between the data of the two samples is summarized in a correlation plot (Figure 2a), with G74, R76, and E79 revealed as outliers, while G74 showed an unusually low *S*^2^ of 0.3. Although not fully quantitative, this is already displayed in the ratio of the NCO cross-peak intensities (Table S5). In an approximation, the cross-peak size reports on the dipolar coupling, with low intensity corresponding to a low *S*^2^ value. The S73C′-G74N cross peak in the OM spectrum (Figure S1), for example, is tiny in comparison to the A99C′-S100N cross peak and also in comparison to the same cross peak in the spectrum of the microcrystalline sample (Figure S1C). Similar effects are seen for N78C′-E79N. The low *S*^2^ values in the β2/L3 region may be explained by dynamics due to structural rearrangements involving the LPS layer. R76, for example, exposes a short *R*_1_ but long *R*_1ρ_, and the SMF analysis of this residue clearly indicates the presence of slow motions (59.8 ± 11.3 ns for YadAM-OM) at a low *S*^2^ = 0.6 ± 0.1, different from the situation in the crystal. The reason for the low-order parameter and slow motion of R76 could be that it is situated in the interior of YadA and involved in the formation of alternative interactions, shuttling between states. Intriguingly, residue E79 in YadAM-OM also shows slow motions (63 ± 16 ns), and one interpretation could be an involvement in contacts with R76.

### Comparison with Molecular Dynamics Simulations

To contribute to a structural interpretation of the observed values, we performed atomistic molecular dynamics (MD) simulations of YadAM (PDB ID: 2LME(14)) embedded in a realistic OM (see Materials and Methods). We ran the simulation in three replicas (2 μs each, yielding a total simulation time of 6 μs) to test the reproducibility of the results (Text S6 and Figure S8). From the trajectories, we calculated the order parameter for each residue of YadAM in the OM model. The average *S*^2^ values are shown in Figure 1e and listed in Table S6, and the *S*^2^ values at different intervals can be found in Table S7 and Figure S9. Notably, the order parameters of the loops exhibited a drop from 120 to 250 ns of the MD simulation but remained relatively stable from 250 ns to 2 μs. This indicates that the loops undergo rapid equilibration on a 100 ns time scale but do not further explore new conformations on the 2 μs time scale. The *S*^2^ values suggest that the α-helical region (residues 25–40) and the strands are the most rigid segments of the protein. However, β2 shows an onset of greater mobility from G72 onward, but it is much less pronounced than observed in the experimental dynamics investigation on YadAM-OM. To understand the unexpected mobility observed in the region following G72, we compared the order parameters obtained from the three 2 μs simulation replicas (Figure S8) and found that replica 2 exhibited the highest order parameter of the three. In this replica, we observed calcium-ion-bridged interactions between E79, very likely supported by potential interactions with nearby phosphate groups of lipids as well as interactions between the head groups of R76 and E79 (Figure 2b). Calcium binding was quantified by calculating the average number of ions trapped within a radius of 3 Å over the course of the simulation (Figure S10). In replica 1, such interactions rarely form. While YadAM in the second replica captures 0.33–0.66 ions/monomer on average, YadAM in the first replica almost never exceeds a value of 0.33. We extracted the residence time of calcium ions by counting how long they remained in contact with the protein after the initial contact was made. The results for each ion that made at least 1 ns of contact are shown in Table S8. Per replica, the longest residence times of the calcium ions were 636, 1880, and 1450 ns. The residence times are consistent with the number of calcium ions trapped by the region spanning residues 75–80 (Figure S10), with more trapped ions corresponding to longer residence times. Furthermore, to address the effect of calcium trapping on lipid diffusion, we measured the diffusion coefficient of lipids along the lateral plane of the membrane (Text S6). The results are shown in Figure S12 and Table S9. Overall, the diffusion coefficients were similar, regardless of whether they were measured near the protein or measured throughout the membrane. However, we do note that there is a contrast between replicas, such that the lipid diffusion coefficients for replica 1 (with the shortest ion residence time) are the highest, while those for replica 2 (with the longest ion residence time) are the lowest among all lipid types. Combined with the above results, we reason that calcium ion trapping may affect the lipid behavior as well.

Upon further inspection, we discovered that, in replica 1, E79’s interaction with Ca^2+^ ions is hindered due to a more persistent interaction with Y49 over the course of the simulation (Figures 2c and S11). On the other hand, R76 and E79 are well situated for interactions with lipid headgroups, increasing their likelihood. To identify if there is any additional mechanism that may be affecting their dynamics, we measured the contact area between two different periplasmic loops (residues 49–54 and residues 75–80) and nearby lipids, as we have done previously.^45^ The results are shown in Figure S13. Interestingly, there is no difference among replicas for contacts between the region 75–80 and the nearby lipids. However, for the first periplasmic loop (residues 49–54), the first replica is involved in more contacts over time, while the second replica is involved in fewer. This finding agrees with the results from Figures 2 and S11; the additional contacts made between Y49 and E79 not only prevent the calcium ions from being trapped but also restrict the first periplasmic loop (that includes Y49) from additional interactions with the lipids. The MD simulations thus suggest a mechanism for the enhanced dynamics in the range of G72-E79 through the exchange between conformational states (Ca^2+^-bound vs R76–E79 interactions), occurring due to the low occupancy of trapped ions as observed for all three replicas. In effect, this may lead to lower-order parameters. Obviously, such interactions can only play a role for YadAM-OM but not for YadAM-Mx, which shows much higher-order parameters for the residues G74 to R76. It is possible that the conditions employed in the simulation and experiments on YadAM-OM differ regarding the effective Ca^2+^ concentrations, explaining the higher mobility in the NMR case. Indeed, the YadAM-OM simulations contained both monovalent and divalent ions, possibly at higher concentrations than the NMR sample.

### Modeling of Global Barrel Motions

The OM of Gram-negative bacteria has unique biophysical properties; hence, it is instructive to model the overall motion of the integrated β-barrel, expected to be in the ns to μs range, via the SMF-GAF approach (Texts S7 and S8).^21,28,29,40^ There, *S*^2^ is replaced by an anisotropic order parameter *S*_μν_^2^ that describes the motion as Gaussian fluctuations along three orthogonal axes, α, β, and γ. Since YadA trimers have cylindrical symmetry (and are located in the membrane plane in the case of YadAM-OM), the number of fit parameters can be reduced to 2 (σ_α_ = σ_∥_, σ_β_ = σ_γ_ = σ_⊥_), with σ_∥_ indicating the principal axis of the cylinder.^28,29,40^ Thus, we obtain two axes, one in-plane axis (σ_⊥_) and one orthogonal to the membrane (σ_∥_) (Figure 1a). In the 3D GAF approach, it is exploited that the N–H bonds fluctuate around the three axes of inertia of the protein. The order parameters depend on the angle between each amide bond vector and the molecular frame and on the amplitudes (σ_∥_, σ_⊥_) of the collective motions. As input data, we used therefore only the two ^15^N relaxation rates (^15^N *R*_1_ and ^15^N *R*_1ρ_) as the specification of an interaction axis via directly bonded protons is required; the ^13^C′ *R*_1ρ_ rates were excluded. The frame of reference was defined by using the available Cartesian coordinates of YadAM-Mx. Since modeling of the global motion was intended, we considered only the relaxation rates that did not reflect local motion, the respective residues mostly located in the strands. For YadAM-Mx, we included the relaxation rates of S44, Y49, N55, F56, A58, A70, G72, S73, G86, V87, 88A, 89Y, M96, A99, S100, and F101 into the calculations. For YadAM-OM, we considered the rates of N43, A58, G72, G86, V87, A88, S100, and F101.

**Figure 3:**
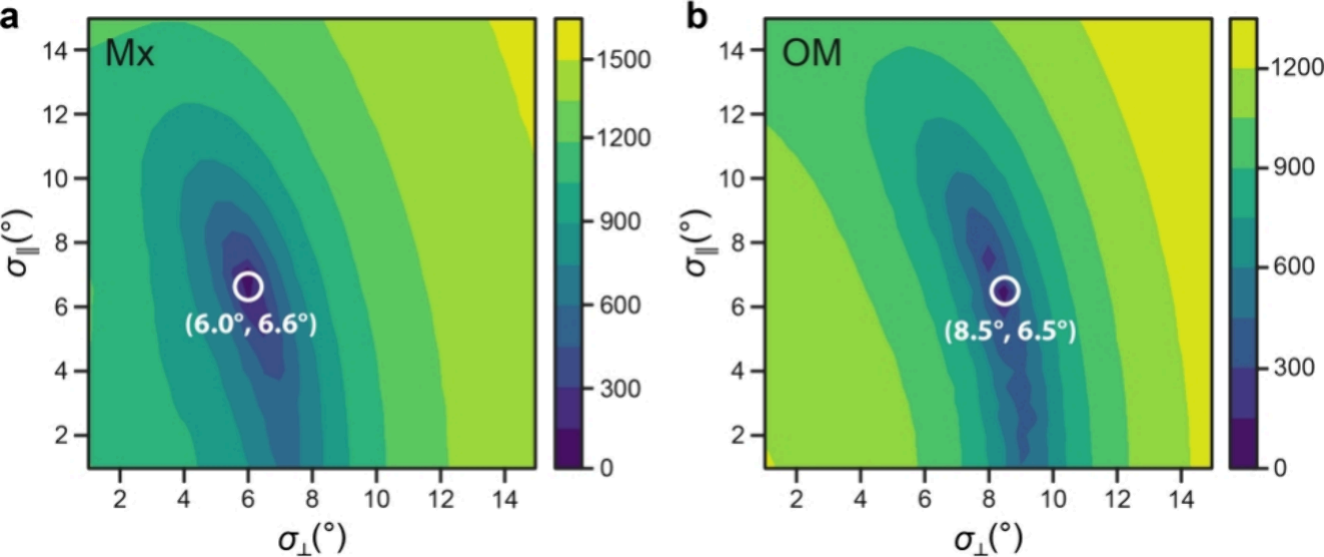
Grid search of the data from the SMF-3D GAF analysis. The grid search results of the SMF-3D GAF analysis for YadAM-Mx and YadAM-OM are shown in (a) and (b), respectively. The grid search analysis was performed on the amplitudes (σ_∥_, σ_⊥_) by stepwise fixing both σ values individually in the range from 1° to 15° in steps of 0.5°. For each pair of fixed amplitudes, we optimized the remaining fit parameters.

A first result is obtained after minimization of the χ^2^ function (Text S7);^40^ the determined fluctuation angles (σ_∥_, σ_⊥_) are (6.4 ± 0.24°, 6.15 ± 0.07°) for Mx and (6.35 ± 0.14°, 8.36 ± 0.4°) for OM. We obtained a correlation time τ_s,μν_ of 41.5 ± 1.1 ns for YadAM-Mx and of 40.8 ± 0.6 ns for YadAM-OM. In order to further analyze if there exists an extended range of motional amplitudes that can describe the experimental data equally well or better, we performed a grid search analysis on the amplitudes (σ_∥_, σ_⊥_) around the initially obtained results (Text S8) by stepwise fixing both values individually in the range from 1° to 15° in steps of 0.5°. For each pair, we optimized the remaining fit parameters. The results are shown in Figure 3a,b for YadAM-Mx and YadAM-OM, respectively, with the white circles on the blue background defining the global minimum around (6.5°, 6°) for YadAM-Mx and (6.5°, 8.5°) for YadAM-OM. Consistently, the grid search yields symmetric fluctuation amplitudes (σ_∥_, σ_⊥_) for YadAM in microcrystals but a larger angular fluctuation amplitude along the transverse axis as compared to the parallel axis for YadAM-OM. The values for the fluctuation angles determined by the grid search match very well with those found after minimization of the χ^2^ function.

## Discussion

This investigation of the anchor domain of YadA in an asymmetric OM environment provides valuable insights into autotransporter dynamics. Our analysis provides evidence of the importance of lipid components in structure stabilization and destabilization. Furthermore, we explored the overall barrel motion by applying SMF-3D GAF approaches, taking anisotropic behavior into account.

Generally speaking, the SMF data of YadAM in OMs show that the four strands of the monomers are the most rigid features, except for the second half of β2, whose *S*^2^ values indicate surprisingly high flexibility. The α-helical stalk and the intracellular loop L3 show also elevated dynamics, but less so L1. A particularly interesting finding is the high mobility of the β2 residues following G72 and of the two β3 residues following the connecting L3. Importantly, G72 itself is not mobile. Conserved Gly–Tyr pairs (mortise-tenon motifs) have been implicated in stabilizing autotransporters and, more generally, OM β-barrel proteins.^17^ The stabilization is relevant both for protein folding intermediates and for the final structure.^17^ Considering the importance of sheet opening and closing processes, it is surprising that here in this case the highest dynamics is found between two internal strands and not within the contact sites of the outer strands β4 and β1 as it would seem more natural. Regarding autotransport, it is expected that the passage of the unfolded passenger domain through the pore has certain space requirements and is promoted by some transient widening of the pore,^14^ for which a potential mechanism is provided here. It can be envisaged that the detected stability of the β1−β4 pairing ensures the intactness of the porin during such passage events.

Intriguingly, the strongest differences between the data obtained on YadAM-OM and YadAM-Mx occur in the two clusters, where the highest values in YadAM-OM are observed. These clusters are the mobility-affected section in the helix of YadA-OM close to the extracellular loops of the barrel, including Y63 in β1, and the G72-β2-L3 region of YadAM-OM.

Our results were supplemented and explained by MD simulations in a realistic OM model, although the *S*^2^ values obtained from the simulation underestimate the mobility of the second half of β2. In particular, there is a considerable difference between the *S*^2^ values of L1 from MD and from the experiment. This could be a limitation of the field strength used, which may not be able to pick up the confinement of residues S44–Y49 inside the barrel. Regardless, the simulations provide a mechanism for disorder in the β2-L3 region through potential Ca^2+^ binding and competing headgroup interaction of R76 and E79. This effect cannot occur in the detergent-containing microcrystals, where less pronounced dynamics are observed. Although the simulation shows a less strong onset of dynamics after G72, it catches the process, in principle, well. However, we note the limitations of the MD simulations, such as not being able to detect the increased flexibility of the ASSA region or the neighboring region, which was predicted by TALOS for both YadAM-Mx and to a larger extent YadAM-OM.^41^

The 3D GAF analysis yields diffusive movements of the β-barrel along two principal axes, one defined by the symmetry axis of the porin (σ_∥_) and the other in-plane (σ_⊥_), describing a combination of a rotation with a rocking component, respectively, that together may produce an overall diffusive tumbling. For both YadAM samples, microcrystalline and embedded into the OM, motions around 41 ns are obtained. YadAM-Mx shows symmetric amplitudes in both dimensions, whereas the amplitude of the rocking component is somewhat larger in the case of YadAM-OM, which might be expected from the higher degrees of freedom in the membrane as compared to the microcrystal. These findings compare well with ssNMR studies on microcrystalline^28^ and membrane proteins^40,29,46^ that all found small-amplitude rocking components of the angular diffusion in the range of tenths to a few hundredths of nanoseconds. In addition, asymmetric amplitudes for the two rotational components (σ_∥_, σ_⊥_) that we observed for YadAM in OM, with a slightly larger amplitude for the in-plane motion (σ_⊥_), were also observed in studies on a bacterial OmpA in lipid membranes.^40^

Our results on the time scales can be compared to a variety of data obtained by other methods such as phosphorescence or luminescence anisotropy of triplet states, absorbance anisotropy, and EPR.^47^ By phosphorescence of triplet states or EPR, the rotational diffusion for proteins in membranes or liposomes is mostly found in the μs to low ms range, with few outliers toward nanoseconds, depending on the membrane and protein system investigated.^47−49^ In our case, we observe a small-amplitude rocking motion of YadAM in native OM that is due to its small deflection supporting rapid motions in the ns range.

In summary, the unusual dynamics observed for the residues following G72 including L3 raises the question of whether this is a feature that could be connected to folding of the β-barrel: to membrane insertion, autotransport, or both. It is generally assumed that protein folding processes start from folding “cores” that pass through different conformations before converging into a final, defined structure.^50,51^ Such folding cores must be inherently flexible to allow such alternative conformations and, maybe counterintuitively, are not always the most stable and rigid part of the final, folded structure. Compared to soluble proteins, membrane proteins have different requirements for folding cores as membrane insertion is part of the folding process.^52^ This is because in this case, interactions with lipids can replace intraprotein hydrophobic interactions to some extent.^53^

For the β-barrel membrane protein OmpX, it has been demonstrated that two regions form the initial secondary structure different from the final β-strand conformation in the otherwise denatured, urea-dissolved polypeptide:^16^ the conserved C-terminal region that later folds into the C-terminal β-strand and that is key to recognition by the BAM complex^54^ and a region around a highly conserved tyrosine residue that interacts with a glycine residue from a neighboring beta-strand to form a “mortoise-tenon motif” that exists both in β-barrel proteins such as OmpX^17^ and in autotransporters.^55^ This latter segment in the OmpX sequence (I73–V82)^16^ not only initiates folding and, presumably, membrane insertion of the protein but also comprises a periplasmic loop in the final, folded OmpX structure.

YadA, as a trimeric autotransporter with highly restricted space in the barrel lumen, does not have a full mortoise-tenon motif, but we observe flexibility in the periplasmic loop region that extends toward the extremely conserved G72 residue. We hypothesize that this periplasmic loop, exactly like in OmpX, needs to adopt alternative conformations during the initiation of membrane insertion or folding, explaining its propensity for mobility in the folded structure. Mutational studies in which the conserved residue G72 has been shown to affect the export and surface display of the YadA passenger domain negatively^12^ suggest a direct role of this residue also in the autotransport process. It is at this point unclear as to how far the two processes of membrane insertion and autotransport are coupled^56,57^ and further research using pulse-chase experiments or similar time-resolved methods will need to be employed to clarify this.

## Experimental Section

### Sample Preparation

Uniformly ^15^N- and ^13^C-labeled YadAM in a microcrystalline form was prepared as previously described.^14,58^ In brief, YadAM was overexpressed in *Escherichia coli* strain BL21 as the host. Membrane protein extraction was facilitated by the detergent poly(ethylene glycol) octyl ether (C8POE, Bachem).^58^ Uniformly labeled samples were produced by using ^13^C, ^15^N-labeled medium (BioExpress, Cambridge Isotopes laboratories) as the carbon source. The protein was purified by separating phases:^59^ the solubilized membrane fraction was mixed with saturated, cold ammonium sulfate solution in the ratio of 3:1 and incubated at 4 °C for 1 h. Then, the upper, detergent-rich phase was collected and dialyzed against a buffer containing 25 mM 3-(*N*-morpholino) propanesulfonic acid, 1 mM ethylenediaminetetraacetic acid, and 1% (w/v) C8POE. The dialyzed sample was centrifuged for 10 min at 10,000 rpm in a Sorvall centrifuge equipped with an SS34 rotor. Afterward, the clear solution was passed over a cation-exchange column (KTA purifier system and an HR10/10 MonoS Column (GE Healthcare)), employing a linear salt gradient. Buffer A was the dialysis buffer, whereas Buffer B contained 1 M NaCl in addition. The protein was eluted at ∼200 mM NaCl. Sodium dodecyl sulfate-polyacrylamide gel electrophoresis was employed for checking its purity. The protein was concentrated by phase separation as described above. Dialysis using tubing with a cutoff of 25 kDa (Roth) served for the removal of salt and excess detergent, while the protein was forming microcrystals that were harvested by centrifugation.

For OM preparations containing YadAM, an *E. coli* knockout strain lacking the major Omps, OmpA, OmpC, OmpF, and LamB (ΔABCF)^42^ was used following a strategy described with some additional modifications to improve the expression system further, regarding both yield and purity of the sample. Briefly, freshly prepared CaCl_2_-competent ΔABCF cells were transformed with pIBA YadAM. Here, we have improved the expression system further, regarding both the yield and purity of the sample. The cells were grown on an LB plate with low salt concentration (5 g/L) and 100 μg/mL ampicillin. A single colony was used to inoculate overnight cultures in LB media (with 5 g/L salt concentration) grown at 26 °C, which were used to inoculate (1:50) 2 L baffled flasks containing 300 mL of Spectra 9 media (Cambridge Isotopes) and grown at 26 °C. Cells were induced with 0.2 μg/mL anhydrotetracycline at an OD_600_ of 0.7 and grown overnight to a final OD_600_ of 2.2–2.5. 600 mL of culture was used for each NMR sample. Cells were harvested by centrifugation at 3000*g* for 20 min and resuspended in PBS buffer (20 mL per 300 mL culture volume) containing 1 mM MgCl_2_ and MnCl_2_, 20 μg/mL DNase, and 0.1 mg/mL lysozyme. Cells were lysed by four passages through a French Press (12–15,000 psi), and nonbroken cells were gently removed at 3000*g* for 3 min to avoid loss of YadAM-containing membrane and centrifuged at 26,000 rpm (Beckman ultracentrifuge with an SW32 rotor) for 1 h to collect the total membrane. The inner membrane was selectively solubilized^60,61^ by resuspending the pellet in 30 mL of 20 mM Tris/HCl pH 8.0 and 1% *N*-laurylsarcosine and incubating for 1 h with gentle rocking at RT. The OM pellet fraction was washed repeatedly with water using ultracentrifugation and resuspended in 20 mM Tris pH 6.0 and 0.02% sodium azide for ssNMR experiments.

### NMR Measurements

The ssNMR measurements were performed on a Bruker Avance-III spectrometer operating at a magnetic field strength of 21.1 T and 60 kHz MAS. The site-specific backbone ^15^N *R*_1_ and ^15^N, ^13^C′ *R*_1ρ_ relaxation measurements were performed by using NCO double cross-polarization (DCP)^62^ and NCO DCP-S3E^63^ (spin state selective) correlation experiments (Figure S2). The data were acquired using a pseudo-3D version of the sequences to ensure constant relative scaling. Further experimental parameters and procedures used in this study are given in the Supporting Information, Text S1 and Table S1. The YadAM-Mx sample was measured at 280 K, and the YadA-OM sample was measured at 286 K. Both temperatures are below 290 K, and there is no phase transition known in that range in OMs. Relevant motional processes in proteins have activation energies of 2–6 (low energy barriers) and 10–35 kJ/mol (high energy barriers).^64^ A difference of 6 K between 280 and 286 K relates then to increases in dynamics by 2–5 and 10–27% for the low and high barrier processes, respectively.

### Model-Free Approaches

The model-free approaches, rate equations, and data fitting procedures27 together with Monte Carlo error estimations are described in the Supporting Information, Texts S3–S5, S7, and S8. MATLAB was used for fitting the relaxation rate to SMF and SMF-3D GAF approaches. The protein preparation employed in this study was fully protonated as well as uniformly ^13^C, ^15^N-labeled. In this case, dipolar couplings involving the proton network influence therefore the signal decays, together with carbon–carbon dipolar couplings and chemical shift anisotropies. We have taken all into account; see the rate equations in Text S3. The experimental and calculated relaxation rates can be correlated by minimizing a χ^2^ function (eq 18 in Text S4, eq 28 in Text S7). The errors associated with relaxation rates, order parameters, and correlation times were obtained by using an iterative Monte Carlo routine. To this end, the optimal fit parameters from each χ^2^ minimalization are first used to back-calculate the experimental relaxation parameters; subsequently, Gaussian noise is added, and the χ^2^ function is minimized again. This was iterated 500 times, yielding the distribution of optimal order parameters and correlation times for each residue or all residues.

### System Generation for MD Simulations

All systems were generated using CHARMM-GUI.^65,66^ The YadAM system was generated on the basis of a previously resolved NMR structure (PDB ID: 2LME(14)). The membrane environment was built primarily based on previously simulated lipid compositions.^67,68^ The LPS used for the outer leaflet of the OM was based on the *E. coli* BL21 strain,^69^ reflecting the experimental membrane composition. The inner leaflet of the membrane was composed of 75% 1-palmitoyl-2-palmitoleoyl-phosphatidylethanolamine, 20% 1-palmitoyl-2-vacenoylphosphatidyl-glycerol, and 5% 1,10-palmitoyl-2,20-vacenoylcardiolipin. LPS was neutralized using divalent ions, with magnesium (Mg^2+^) for lipid A and calcium (Ca^2+^) for the LPS core sugars. The system was solvated and ionized to a concentration of 0.15 M of potassium chloride (KCl).

### MD Simulations

All-atom MD simulations were performed using NAMD3^70^ along with the CHARMM36m force field for proteins^71,72^ and the CHARMM36 force field for lipids^73^ along with TIP3P water.^74^ All simulations were performed under periodic boundary conditions with the cutoff at 12 Å for short-range electrostatic and Lennard-Jones interactions. A force-based switching function starting at 10 Å was also used. The particle-mesh Ewald method^75^ with a grid spacing of maximum 1 Å was used for calculation of long-range electrostatic interactions. Bonds between a heavy atom and a hydrogen atom were maintained to be rigid, while all other bonds remained flexible. Unless otherwise stated, each system was equilibrated for 2 μs under an isothermal–isobaric ensemble (*NPT*) at 310 K and 1 bar, with a time step of 4 fs with hydrogen mass repartitioning.^76,77^ A Langevin thermostat with a damping coefficient of 1 ps^–1^ was used for temperature control, and a Langevin piston with a period of 0.1 ps and a decay of 0.05 ps was used for pressure control. The production phase for each system included three replicas in order to check the reproducibility of our results.^78^ The total simulation time was 6 μs (2 μs × 3 replicas). The simulations were analyzed using the molecular visualization and analysis software Visual Molecular Dynamics.^79^ Order parameters and relaxation rates were calculated according to Text S6.

## Data Availability

All data needed to evaluate the conclusions in the paper are present in the paper and/or in the Supporting Information.

## Supporting Information Available

Parameters of the NMR experiments, error calculation, overview of motional models and relaxation theory, description of SMF formalism, methodology followed for back-calculation of relaxation rates, methodology to calculate order parameters and relaxation rates from MD simulations, description of 3D GAF formalism, grid search analysis for the 3D GAF model; supplementary figures of 2D NCO and NCA correlation spectra of both YadAM-Mx and YadAM-OM, NMR pulse sequences, ^15^N *R*_1_, ^15^N *R*_1ρ_, and ^13^C′ *R*_1ρ_ decay curves of both samples, back-calculated rates from the SMF model for YadAM-Mx and YadAM-OM, MD-derived order parameters for YadAM-OM from different replicas and over different time intervals, plots over time of trapped calcium ions and contact area between residues Y49 and E79 in MD simulations of YadAM-OM, mean squared displacement of different lipids over different time intervals, and contact between periplasmic loops and lipids over time; supplementary tables detailing experimental parameters used to acquire the NMR spectra, ^15^N *R*_1_, ^15^N *R*_1ρ_, and ^13^C′ *R*_1ρ_ relaxation rates of YadAM-Mx and YadAM-OM, fit parameters for SMF calculations of both YadAM-Mx and YadAM-OM, back-calculated rates for all SMF results, peak volumes of YadAM-Mx and YadAM-OM signals, MD-derived order parameters for NH vectors of YadAM-OM, MD-derived order parameters of YadAM-OM at different time intervals, residence time of calcium ions interacting with the protein, and diffusion coefficient of lipids overall and near the protein (PDF)
