# Supplementary material for: Probing the Dynamics of Yersinia Adhesin A (YadA) in Outer Membranes Hints at Requirements for β-Barrel Membrane Insertion
Source: J Am Chem Soc. 2025 Feb 27;147(10):8618–28. doi: 10.1021/jacs.4c17726 (PMC11912334; doi:10.1021/jacs.4c17726)
Supplement: Supplementary file 1 — ja4c17726_si_001.pdf [file ja4c17726_si_001.pdf]

Supporting Information

Probing the dynamics of Yersinia adhesin A (YadA) in outer membranes hints at requirements for β-barrel membrane insertion

Jayasubba Reddy Yarava[1,7], Marcella Orwick-Rydmark[2], David Ryoo[3], Albert Hofstetter[4], James C. Gumbart[5], Michael Habeck[6], Barth-Jan van Rossum[1], Dirk Linke[2] and Hartmut Oschkinat[1] *

[1]Leibniz-Forschungsinstitut für Molekulare Pharmakologie, Robert-Rössle-Straße 10, 13125 Berlin, and Freie Universität Berlin, Takustraße 3, 14195 Berlin, Germany

[2]Department of Biosciences, University of Oslo, P.O.Box 1066 Blindern, 0316 Oslo, Norway

[3]Interdisciplinary Bioengineering Graduate Program, Georgia Institute of Technology, Atlanta, GA 30332, United States of America

[4]Department of Chemistry and Applied Biosciences, ETH Zurich, Vladimir-Prelog-Weg 2, 8093 Zurich, Switzerland.

[5]School of Physics, Georgia Institute of Technology, Atlanta, GA 30332, United States of America

[6]Microscopic Image Analysis group, Jena University Hospital, Am Klinikum 1, 07747, Jena, Germany

[7]Current Address: Department of Chemistry, Michigan State University, East Lansing, MI-48824, United States of America

KEYWORDS. protein dynamics, membrane protein, magic-angle spinning, simple-model free, relaxation, autotransporter, microcrystals, native-membranes, outer membranes

Index

List of text

Text S1. NMR experiments

Text S2. Error Calculation

Text S3. Motional models - Relaxation theory

Text S4. Simple model free (SMF) formalism

Text S5. Validating determined dynamical parameters through the back calculation of relaxation rates

Text S6. Order parameter and relaxation rate calculation from MD simulation

Text S7. 3D Gaussian axial fluctuations model (3D GAF) formalism

Text S8. Grid search analysis for 3D GAF model

List of Figures

Figure S1. 2D NCO and NCA correlation spectra of YadAM-Mx and YadAM-OM

Figure S2. 2D NCO and 2D NCO-S3E pulse sequences for the relaxation measurements

Figure S3. ^{15}N R_1 decay curves of YadAM-Mx and YadAM-OM

Figure S4. ^{15}N $R_{1\rho}$ decay curves of YadAM-Mx and YadAM-OM

Figure S5. ^{13}C' $R_{1\rho}$ decay curves of YadAM-Mx and YadAM-OM

Figure S6. Back-calculated rates from SMF model for YadAM-Mx

Figure S7. Back-calculated rates from SMF model for YadAM-OM

Figure S8. MD-derived order parameters for YadAM-OM from three 2-μs simulation replicas

Figure S9. MD-derived order parameters for YadAM-OM from different portions of the 2-μs simulations

Figure S10. Calcium ions trapped in MD simulations for YadAM-OM

Figure S11. Contact area between residues Y49 and E79 in simulations of YadAM-OM

Figure S12. Mean squared displacement (MSD) different lipids over different time intervals

Figure S13. Contact between periplasmic loops and lipids over time

List of Tables

Table S1. Experimental parameters used to acquire the NMR spectra

Table S2. ^{15}N R_1, ^{15}N $R_{1\rho}$, and ^{13}C' $R_{1\rho}$ relaxation rates of YadAM-Mx and YadAM-OM

Table S3. Fit parameters for SMF model applied to YadAM-Mx and YadAM-OM

Table S4. Back-calculated rates from SMF model for YadAM-Mx and YadAM-OM

Table S5. Peak volumes of YadAM-Mx and OM signals

Table S6. MD-derived order parameters of YadAM-OM for NH vector.

Table S7. MD-derived order parameters of YadAM-OM at different time intervals

Table S8. Residence time of calcium ions interacting with the protein

Table S9. Diffusion coefficient of lipids overall and near the protein

Text S1: NMR Experiments

Solid-state NMR measurements were performed on a Bruker Avance-III spectrometer operating at a magnetic field strength of 21.1 T. A Bruker 1.3 mm triple resonance MAS probe was used, operated at a sample spinning frequency of 60 kHz. A sample temperature of 280 K for YadAM-Mx and 286 K for YadAM-OM was maintained during the course of the experiments. The chemical shifts of YadAM-Mx were referenced with respect to the previously reported chemical shifts[1] (BMRB entry ID:18108). The chemical shifts of YadAM-OM were referenced by superimposing the 2D NCO correlation spectrum with the YadAM-Mx NCO spectrum. The 2D NCO double CP (DCP) spectra of YadAM-Mx and YadAM-OM are shown in Figure S1.

An external deuterium capsule was used to lock and compensate for the field drift. Site-specific backbone ^{15}N R_1 and ^{15}N, ^{13}C' $R_{1\rho}$ relaxation measurements were performed by using NCO double cross-polarization[2] and spin-state selective NCO DCP-S3E[3] correlation experiments. The pulse sequences are shown in Figure S2. The ^{15}N R_1 rates were measured using a standard NCO DCP experiment. A $\pi/2$-delay-$\pi/2$ block was inserted just before the t_1 increment on the ^{15}N channel, and this delay was incremented in a series of experiments (Figure S2 A)[4]. For the ^{15}N $R_{1\rho}$ relaxation measurements, a ^{15}N spin-lock pulse was applied after the first CP (Figure S2 B). The duration of the pulse was incremented in a series of experiments. Similarly, for the ^{13}C' $R_{1\rho}$ measurements, a ^{13}C spin-lock pulse of variable length was used after the second CP on the ^{13}C channel (Figure S2 C). All data were acquired as a pseudo 3D experiment to ensure constant relative scaling. The data of YadAM-Mx and YadAM-OM were processed using Topspin; peak volumes were extracted using the CcpNmr software[5].

For YadAM-Mx, the ^{15}N R_1 relaxation rates were measured using a series of recovery delays of 0.5, 1, 5, 10, 16, 23, 32 and 42 s (Figure S2 A). For each 2D spectrum, a total of 192 t_1 increments were used. For each t_1 increment, a total of 16 transients were coadded with a recycle delay of 3 s. Data were acquired using the States method. The total experimental time was 5 days 11 h. To improve the resolution for the YadAM-Mx sample, 2D spin-state selective DCP-S3E sequences were used. For the S3E experiments, data were acquired in interleaved acquisition mode which doubled the experiment time. Since the ^{15}N R_1 measurements for YadAM-Mx require a long recycle delays of up to 42 s, doubling the experiment time by using a DCP-S3E sequence was not practical in that case and instead spectra were acquired with long t_1 delays (Figure S2 A). The pseudo-3D DCP-S3E pulse sequence used for measuring ^{15}N $R_{1\rho}$ relaxation rates is shown in Figure S2 D. An RF field strength of 17 kHz was used on the ^{15}N channel during time variable spinlock period. A series of experiments were measured with spinlock times of 0.5, 20, 48, 101, 164, 225, 275 and 315 ms. For each 2D spectrum, 128 t_1 increments were used. For each t_1 increment, a total of 32 transients were coadded with a recycle delay of 3 s. Data were acquired in interleaved acquisition mode using the States method[6]. The total experimental time was 2 days 6.6 h. The ^{13}C' $R_{1\rho}$ relaxation rates were measured using the pseudo-3D DCP-S3E pulse sequence shown in Figure S2 E. An RF field strength of 17 kHz was used on the ^{13}C' channel during the time variable spinlock period. A series of experiments were measured with spinlock times of 0.5, 10, 20, 36, 47 and 62 ms. A total of 64 transients were used for each of 96 t_1 points. Data were acquired in interleaved acquisition mode with the States method. The total experimental time was 2 days 13.4 h.

For the YadAM-OM sample, the ^{15}N R_1 measurements were performed using a series of recovery delays of 0.5, 2, 5, 10, 14, 18, 23 and 32 s (Figure S2 A). A total number of 112 t_1 points were used to acquire the spectrum. For each t_1 increment, a total of 64 transients were coadded with a recycle delay of 3 s. Data were acquired with the States method. The total experimental time was 10 days 16 h. For the ^{15}N $R_{1\rho}$ relaxation measurements, a 17 kHz RF

field strength was used for variable length spinlock period. A series of experiments were measured using pulse sequence shown in Figure S2 B with spinlock times of 0.5, 5, 10, 20, 40, 60, 80 and 120 ms. A total of 128 scans were used for each of 128 t_1 increments to acquire the 2D spectrum. The total experimental time was 4 days 13 h. For the ^{13}C' $R_{1\rho}$ relaxation measurements, a 17 kHz RF field strength was used during spinlock period on the ^{13}C channel. A series of experiments were measured using pulse sequence shown in Figure S2 C with spinlock times of 0.5, 2.5, 5, 7.5, 10, 15, 20 and 25 ms. A total of 48 scans were used for each of the 90 t_1 increments to acquire the spectrum. The total experimental time was 28.8 h. Other experimental details are listed in Table S1.

In summary, for the YadAM-Mx sample, data collection for the relaxation experiments took 19 days 7 hours, while for the YadAM-OM sample, it took 16 days and 8 hours. Hence the total experimental time for collecting relaxation data from both samples was 35 days and 15 hours.

For the 2D NCA spectrum, a total of 160 t_1 increments were acquired. For each t_1 increment, 64 transients were co-added with a recycle delay of 3 s. The ^{1}H-^{15}N CP contact time was set to 1.1 ms, while the ^{15}N-^{13}CA CP contact time was 9 ms. Data were acquired using the States-TPPI method, with a total experimental time of 8.5 h.

Text S2: Error Calculation

Spectra analysis, peak picking and peak volume calculation was performed with version 2.4.2 of CcpNmr[5]. For the error estimation of the fits of the relaxation data, we used the following procedure. We first picked all “noise” peaks for a region of the spectra that is essentially signal free (^{13}C: 130-110 ppm, ^{15}N: full spectral width). For these (>1500) peaks, the positive or negative volume was determined using the ‘box sum’ setting with standard parameters. Subsequently, the mean and standard deviation of the volume distribution was calculated. As the distribution of peak volumes does not follow a normal distribution (peaks with no volumes are, correctly, not picked by the algorithm) a correction was applied. For this, a histogram with 51 bins of the peak volumes normalized to the largest one was calculated. Afterwards, a scaled normal distribution

$$y = \frac{1}{\sigma\sqrt{2\pi}} e^{-\frac{1}{2}\left(\frac{x-\bar{x}}{\sigma}\right)^2}$$

was fitted to the centres of bins 4-17 and 34-48 (indicated with the red dots in figures below) by a least-squares approach using the *curve_fit* function of the python package *scipy.optimize*.

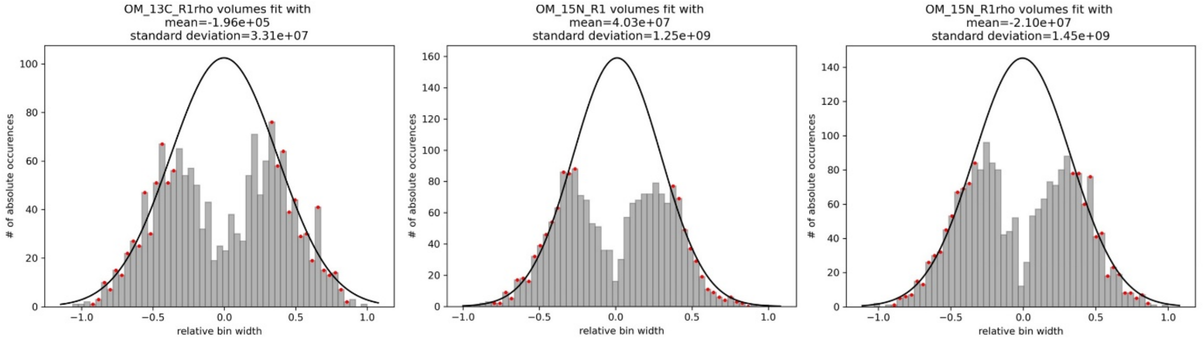

Text S3: Motional Models – Relaxation Theory

Different nuclear spin interactions contribute to the spin relaxation process. The rate equations for ^{15}N and $^{13}C'$ used here are explained in Lamley et al.[4] and Öster et al.[7] The main contributions to ^{15}N R_1 and $R_{1\rho}$ relaxation come from the ^{15}N-^{1}H dipolar interactions and the ^{15}N CSA. Since the proteins in our study are uniformly labelled both with ^{13}C and ^{15}N, there will also be contributions from ^{15}N-$^{13}C_{\alpha}$ and ^{15}N-$^{13}C'$ dipolar couplings that are at least about four times weaker than the ^{15}N-^{1}H couplings. In addition, the proteins are fully protonated so there will be ^{15}N-^{1}H dipolar couplings with both the directly attached proton as well as with remote ^{1}H spins. The ^{15}N $R_{1\rho}$ relaxation rates are sensitive to the spin-lock frequency and MAS rate. The $^{13}C'$ relaxation rates are similarly influenced by interactions with (distant) protons and CSA as well as by directly bonded $^{13}C_{\alpha}$.

(i) The contribution of the ^{15}N-^{1}H dipolar interactions to ^{15}N R_1 is given by:

$$R_{1,\mathrm{NH}} = \frac{1}{10}\left(\frac{\mu_0}{4\pi}\frac{\hbar\gamma_\mathrm{H}\gamma_\mathrm{N}}{r_\mathrm{NH}^3}\right)^2 \left(J_0(\omega_\mathrm{H}-\omega_\mathrm{N}) + 3J_1(\omega_\mathrm{N}) + 6J_2(\omega_\mathrm{H}+\omega_\mathrm{N})\right) \quad (1)$$

Here μ_0 is the magnetic permeability in vacuum, γ the gyromagnetic ratio for the specified nucleus and $\hbar$ is Planck's constant. For the contribution from directly attached protons to ^{15}N we took a distance r_NH of 1.02 Å and for the nearest remote proton we took an r_NH of 1.8 Å. $J(\omega)$ is the spectral density function which will be modelled according to the SMF and 3D-GAF approaches.

(ii) Contribution of the CSA to ^{15}N R_1:

$$R_{1,\mathrm{N,CSA}} = \frac{2}{15}\omega_\mathrm{N}^2(\Delta\sigma^2)J_1(\omega_\mathrm{N}) \quad (2)$$

For the ^{15}N CSA we used $\Delta\sigma$= 160 ppm.

(iii) Contribution of the $^{13}C_{\alpha}$ -^{15}N dipolar interactions to ^{15}N R_1

$$R_{1,\mathrm{C\alpha N}} = \frac{1}{10}\left(\frac{\mu_0}{4\pi}\frac{\hbar\gamma_\mathrm{C}\gamma_\mathrm{N}}{r_\mathrm{C\alpha N}^3}\right)^2 \left(J_0(\omega_\mathrm{C}-\omega_\mathrm{N}) + 3J_1(\omega_\mathrm{N}) + 6J_2(\omega_\mathrm{C}+\omega_\mathrm{N})\right) \quad (3)$$

To account for the dipolar coupling between the $^{13}C_{\alpha}$ and ^{15}N nuclei we took a CN distance of 1.46 Å.

(iv) Contribution of the $^{13}C'$-^{15}N dipolar interactions to ^{15}N R_1

$$R_{1,\mathrm{C'N}} = \frac{1}{10}\left(\frac{\mu_0}{4\pi}\frac{\hbar\gamma_\mathrm{C}\gamma_\mathrm{N}}{r_\mathrm{C'N}^3}\right)^2 \left(J_0(\omega_\mathrm{C}-\omega_\mathrm{N}) + 3J_1(\omega_\mathrm{N}) + 6J_2(\omega_\mathrm{C}+\omega_\mathrm{N})\right) \quad (4)$$

To account for the dipolar coupling between the $^{13}C'$ and ^{15}N nuclei, we employed a CN distance of 1.33 Å.

The individual spectral density terms combine to the following rate equations for the ^{15}N $R_{1\rho}$ and $^{13}C'$ $R_{1\rho}$:

(v) Contribution of the ^{15}N-^{1}H dipolar interactions to ^{15}N $R_{1\rho}$

$$R_{1\rho,\mathrm{NH}} = \frac{1}{20}\,(\frac{\mu_0}{4\pi}\,\frac{\hbar\gamma_\mathrm{H}\gamma_\mathrm{N}}{r_\mathrm{NH}^3})^2\,\Big(\frac{2}{3}J_0(\omega_1+2\omega_r)+\frac{2}{3}J_0(\omega_1-2\omega_r)+\frac{4}{3}J_0(\omega_1+\omega_r)+\frac{4}{3}J_0(\omega_1-\omega_r)+3J_1(\omega_\mathrm{N})+J_0(\omega_\mathrm{H}-\omega_\mathrm{N})+6J_1(\omega_\mathrm{H})+6J_2(\omega_\mathrm{H}+\omega_\mathrm{N})\Big) \quad (5)$$

In our experiments, ω_1= 17 kHz and ω_r = 60 kHz

(vi) Contribution of the CSA to ^{15}N $R_{1\rho}$

$$R_{1\rho,\mathrm{N,CSA}} = \frac{1}{45}\,\omega_\mathrm{N}^2(\,\Delta\sigma^2)\,\Big(\frac{2}{3}J_0(\omega_1+2\omega_r)+\frac{2}{3}J_0(\omega_1-2\omega_r)+\frac{4}{3}J_0(\omega_1+\omega_r)+\frac{4}{3}J_0(\omega_1-\omega_r)+3J_1(\omega_\mathrm{N})\Big) \quad (6)$$

As above, for the ^{15}N CSA we used $\Delta\sigma$= 160 ppm.

(vii) Contribution of the ^{13}C' -^{15}N dipolar interactions to ^{15}N $R_{1\rho}$

$$R_{1\rho,\mathrm{C'N}} = \frac{1}{20}\,(\frac{\mu_0}{4\pi}\,\frac{\hbar\gamma_\mathrm{C}\gamma_\mathrm{N}}{r_\mathrm{C'N}^3})^2\,(\frac{2}{3}J_0(\omega_1+2\omega_r)+\frac{2}{3}J_0(\omega_1-2\omega_r)+\frac{4}{3}J_0(\omega_1+\omega_r)+\frac{4}{3}J_0(\omega_1-\omega_r)+3J_1(\omega_\mathrm{N})+J_0(\omega_\mathrm{C}-\omega_\mathrm{N})+6J_1(\omega_\mathrm{C})+6J_2(\omega_\mathrm{C}+\omega_\mathrm{N})) \quad (7)$$

As above, we employed a CN distance of 1.33 Å to account for the dipolar coupling between the ^{13}C' and ^{15}N nuclei.

(viii) Contribution of the $^{13}C_\alpha$ -^{15}N dipolar interactions to ^{15}N $R_{1\rho}$

$$R_{1\rho,\mathrm{C\alpha N}} = \frac{1}{20}\,(\frac{\mu_0}{4\pi}\,\frac{\hbar\gamma_\mathrm{C}\gamma_\mathrm{N}}{r_\mathrm{C\alpha N}^3})^2\,(\frac{2}{3}J_0(\omega_1+2\omega_r)+\frac{2}{3}J_0(\omega_1-2\omega_r)+\frac{4}{3}J_0(\omega_1+\omega_r)+\frac{4}{3}J_0(\omega_1-\omega_r)+3J_1(\omega_\mathrm{N})+J_0(\omega_\mathrm{C}-\omega_\mathrm{N})+6J_1(\omega_\mathrm{C})+6J_2(\omega_\mathrm{C}+\omega_\mathrm{N})) \quad (8)$$

We used a CN distance of 1.46 Å to account for the dipolar coupling between the $^{13}C_\alpha$ and ^{15}N nuclei.

(ix) Contribution of the CSA to ^{13}C' $R_{1\rho}$

$$R_{1\rho,C',CSA} = \frac{1}{45}\,\omega_C^2(\Delta\sigma^2)(\frac{2}{3}J_0(\omega_1+2\omega_r)+\frac{2}{3}J_0(\omega_1-2\omega_r)+\frac{4}{3}J_0(\omega_1+\omega_r)+\frac{4}{3}J_0(\omega_1-\omega_r)+3J_1(\omega_C) \quad (9)$$

For the ^{13}C' CSA we employed $\Delta\sigma$= 172 ppm.

(x) Contribution of the ^{13}C -^{13}C' dipolar interactions to ^{13}C' $R_{1\rho}$

$$R_{1\rho,\mathrm{C'C\alpha}} = \frac{1}{20}\left(\frac{\mu_0}{4\pi}\frac{\hbar\gamma_{\mathrm{C}}\gamma_{\mathrm{C}}}{r^3_{\mathrm{C'C\alpha}}}\right)^2\left(\frac{2}{3}J_0(\omega_1+2\omega_r)+\frac{2}{3}J_0(\omega_1-2\omega_r)+\frac{4}{3}J_0(\omega_1+\omega_r)+\frac{4}{3}J_0(\omega_1-\omega_r)+J_0(\omega_{\mathrm{C'}}-\omega_{\mathrm{C\alpha}})+9J_1(\omega_{\mathrm{C}})+6J_2(2\omega_{\mathrm{C}})\right) \quad (10)$$

We used a difference $\omega_{\mathrm{C'}}-\omega_{\mathrm{C\alpha}}$ of 120 ppm.

(xi) Contribution of the ^{1}H-^{13}C' dipolar interactions to ^{13}C' $R_{1\rho}$

$$R_{1\rho,\mathrm{C'H}} = \frac{1}{20}\left(\frac{\mu_0}{4\pi}\frac{\hbar\gamma_{\mathrm{H}}\gamma_{\mathrm{C}}}{r^3_{\mathrm{C'H}}}\right)^2\left(\frac{2}{3}J_0(\omega_1+2\omega_r)+\frac{2}{3}J_0(\omega_1-2\omega_r)+\frac{4}{3}J_0(\omega_1+\omega_r)+\frac{4}{3}J_0(\omega_1-\omega_r)+3J_1(\omega_{\mathrm{C}})+J_0(\omega_{\mathrm{H}}-\omega_{\mathrm{C}})+6J_1(\omega_{\mathrm{H}})+6J_2(\omega_{\mathrm{H}}+\omega_{\mathrm{C}})\right) \quad (11)$$

We employed a CH distance of 2.04 Å to account for the dipolar coupling between the ^{13}C' and nearby ^{1}H nuclei.

(xii) Contribution of the ^{13}C'-^{15}N dipolar interactions to ^{13}C' $R_{1\rho}$

$$R_{1\rho,\mathrm{C'N}} = \frac{1}{20}\left(\frac{\mu_0}{4\pi}\frac{\hbar\gamma_{\mathrm{C}}\gamma_{\mathrm{N}}}{r^3_{\mathrm{C'N}}}\right)^2\left(\frac{2}{3}J_0(\omega_1+2\omega_r)+\frac{2}{3}J_0(\omega_1-2\omega_r)+\frac{4}{3}J_0(\omega_1+\omega_r)+\frac{4}{3}J_0(\omega_1-\omega_r)+3J_1(\omega_{\mathrm{C}})+J_0(\omega_{\mathrm{C}}-\omega_{\mathrm{N}})+6J_1(\omega_{\mathrm{N}})+6J_2(\omega_{\mathrm{C}}+\omega_{\mathrm{N}})\right) \quad (12)$$

As above, we used a CN distance of 1.33 Å to account for the dipolar coupling between the ^{13}C' and ^{15}N nuclei.

Text S4: Simple model free (SMF) formalism

The spectral density is a function of the order parameter S^2, representing the amplitude of motion, and the effective correlation time τ_{eff}, which for solid-state NMR is given as[8,9]:

$$J(\omega) = (1-S^2)\frac{\tau_{\mathrm{eff}}}{1+(\omega\tau_{\mathrm{eff}})^2} \quad (13)$$

For all different contributions to the relaxation processes (Eqs. 1-12, text S3), the density functions $J_n(\omega)$ are replaced with the density function in Eq. 13. In essence, this makes the various rate equations a function of S^2, τ_{eff} and ω.

The ^{15}N R_1 relaxation is given by sum of the different contributions (Eqs. 1-4), as

$$^{15}\mathrm{N}\ R_1 = R_{1,\mathrm{NH}} + R_{1,\mathrm{N,CSA}} + R_{1,\mathrm{C\alpha N}} + R_{1,\mathrm{C'N}} \quad (14)$$

Likewise, the ^{15}N $R_{1\rho}$ and ^{13}C' $R_{1\rho}$ are obtained by summing the respective contributions (Eqs. 5-12),

$$^{15}\mathrm{N}\ R_{1\rho} = R_{1\rho,\mathrm{NH}} + R_{1\rho,\mathrm{N,CSA}} + R_{1\rho,\mathrm{C\alpha N}} + R_{1\rho,\mathrm{C'N}} \quad (15)$$

^{13}C' $R_{1\rho} = R_{1\rho,\,\mathrm{C'H}} + R_{1\rho,\,\mathrm{C',\,CSA}} + R_{1\rho,\,\mathrm{C'N}} + R_{1\rho,\,\mathrm{C'C\alpha}}$ (16)

For the fitting procedure, the experimental intensities $I_{Rm}^{\mathrm{expt}}(t_k)$ were first obtained from the measured decay curves. Here $R_m = R_1 \ or \ R_{1\rho}$, and t_k is the time series of recovery delays or series of variable spin-lock times. The calculated intensities were obtained using the following equation:

$$I_{Rm}^{\mathrm{calc}}(t_k) = I_{Rm}\exp\left(-R_m(S^2, \tau_{\mathrm{eff}}, \omega)t_k\right) \quad (17)$$

The experimental and calculated intensities were correlated by minimizing the χ^2 function

$$\chi^2 = \frac{1}{N}\sum_{k=1}^{N}\left(\frac{I_{15\mathrm{NR1}}^{\mathrm{expt}}(t_k) - I_{15\mathrm{NR1}}^{\mathrm{calc}}(t_k)}{\sigma_{15\mathrm{NR1,expt}}^2}\right)^2 + \frac{1}{N}\sum_{k=1}^{N}\left(\frac{I_{15\mathrm{NR1\rho}}^{\mathrm{expt}}(t_k) - I_{15\mathrm{NR1\rho}}^{\mathrm{calc}}(t_k)}{\sigma_{15\mathrm{NR1\rho,expt}}^2}\right)^2 + \frac{1}{N}\sum_{k=1}^{N}\left(\frac{I_{13\mathrm{C'R1\rho}}^{\mathrm{expt}}(t_k) - I_{13\mathrm{C'R1\rho}}^{\mathrm{calc}}(t_k)}{\sigma_{13\mathrm{C'R1\rho,expt}}^2}\right)^2 \quad (18)$$

Here, σ_{expt}^2 is the experimental noise and N is number of experimental relaxation data points.

In the SMF model, it is assumed that the local motions are isotropic, with one correlation time and one order parameter per residue of the protein. As input for the SMF model, we use experimentally determined ^{15}N R_1, ^{15}N $R_{1\rho}$ and ^{13}C' $R_{1\rho}$ rates as fit parameters. The relaxation rates can be calculated from the order parameter S^2 and effective correlation time τ_{eff} using Eq. 17. The experimental and calculated intensities can be correlated by minimizing the χ^2 function (Eq. 18).

The order parameter S^2 and effective correlation time τ_{eff} are then obtained using an iterative Monte Carlo routine. To this end, χ^2 is minimized to find optimal fit parameters. Subsequently, Gaussian noise is added to the original experimental relaxation parameters and the χ^2 function is minimized again[4]. This procedure was iterated 500 times, yielding the distribution of optimal order parameters and effective correlation times for the residues for which experimental data were available. The determined fit parameters are listed in Tables S2 and S3.

Text S5: Validating determined dynamical parameters through the back calculation of relaxation rates

The dynamical parameters obtained from SMF motional model was cross-validated by back calculating the rates. Initially, a Monte-Carlo simulation was performed, which provided not only the mean of our model parameters but also their standard deviations and parameter distributions. This facilitated an evolution of the uncertainty linked to our model parameters in the presence of experimental noise. Subsequently, the distribution of model parameters was utilized to back calculate the relaxation rates by minimizing the χ^2 function, allowing for the estimation of the associated uncertainties in the calculated rates.

In the case of the SMF model, the resulting back-calculated rates (^{15}N R_1, ^{15}N $R_{1\rho}$ and ^{13}C' $R_{1\rho}$) closely match the experimentally determined rates for YadAM-Mx (Figure S6) and YadAM-OM (Figure S7), detailed in Table S4. Although most rates in the SMF model are comparable,

some differences are noticed in ^{15}N $R_{1\rho}$ rates, for example for the residues of G74, Y75, R76, E79, V81 and A82. The experimental rates for these residues are higher which may indicate the existence of slow local motions. The residue-specific analysis of the relaxation data in this SMF model reveals a good agreement between the back-calculated rates and the experimentally determined rates.

Text S6: Order parameter and relaxation rate calculation from MD simulation

The order parameter and correlation function in different time intervals for each residue were calculated from our simulations following previously published procedures[10-13] based on a model-free approach[8,9]. First, all the frames of the simulations were aligned using the C_α atoms of all residues to eliminate the global motion. Then, for each residue, the vector between the backbone nitrogen and its covalently bound hydrogen, $n(t)$, was calculated. Using the vector's x, y, and z components, the order parameter, S^2, was calculated according to the equation

$$S^2 = C_\mathrm{I}(\infty) = \frac{4\pi}{5}\sum_{m=-2}^{2}\langle Y_{2m}\rangle\langle Y_{2m}^*\rangle = \frac{3}{2}\left(\langle x^2\rangle^2 + \langle y^2\rangle^2 + \langle z^2\rangle^2 + 2\langle xy\rangle^2 + 2\langle xz\rangle^2 + 2\langle yz\rangle^2\right) - \frac{1}{2} \tag{19}$$

where < > denotes the averages over the simulation trajectory. The correlation function was calculated as follows:

$$C_{NH}(\tau) = \langle P_2[\vec{n}(t+\tau)\cdot\vec{n}(t)]\rangle_t = A_0 + \sum_{i=1}^{3} A_i e^{-\tau/\tau_i} \tag{20}$$

where $P_2(x) = 1/2\ (3x^2 - 1)$ and the brackets ($< >_t$) indicate a trajectory average over a sliding window beginning at all possible initial times t. The A_0 is the plateau value of the correlation function, such that:

$$C_{NH}(\infty) = S^2 = A_0 \tag{21}$$

After calculating the correlation function for each residue over time, a sum of three exponentials was fit as indicated in Eq. 20. The parameters from this fit are used to calculate the spectral density J:

$$\begin{aligned} J(\omega) &= 2\int_0^\infty C(t)\cos(\omega t)\mathrm{d}t \\ &= \frac{A_0 2\tau_\mathrm{c}}{1+(\omega\tau_\mathrm{c})^2} + \sum_{i=1}^{3}\frac{A_i 2\tau_{i\mathrm{eff}}}{1+(\omega\tau_{i\mathrm{eff}})^2} \end{aligned} \tag{22}$$

where

$$\sum_{i=0}^{3} A_i = 1 \quad A_i \geq 0, \tau_i \geq 0 \tag{23}$$

The correlation time τ_C was calculated according to the equations in Zhang et al.[10]

$$\tau_c = \frac{1}{1-S^2}\int_0^{T_{conv}} \left(C_{NH}(t) - S^2\right) \mathrm{d}t. \tag{24}$$

To calculate the convergence time T_{conv}, we first took the average of the last 5 ns from the correlation function as the long-time limit $C_{NH}(\infty)$. This order parameter and that calculated in Eq. 19 are comparable, although they are not expected to be identical[11]; T_{conv} was taken as the time at which the correlation first comes within 0.0005 of $C_{NH}(\infty)$ or if it reaches 0. The ^{15}N R_1 and ^{15}N $R_{1\rho}$ were calculated using Eqns. 1 and 5, accounting for both major and minor contributions.

The diffusion coefficient was calculated as done previously[14,15]. Per replica, we used the entire 2 µs of the simulation with intervals up to 1 µs to calculate the mean squared displacement (MSD) of the lipids based on our previous study[16]. Then, we plotted the MSD values from 100 ns to 350 ns and carried out a linear regression to determine the diffusion coefficient (D) for each lipid. Because the lipid movement is restricted laterally (that is, 2D), the diffusion coefficient can be expressed as D = MSD / 4t.

Text S7: 3D Gaussian axial fluctuations model (3D GAF) formalism

The 3D-Gaussian axial fluctuation (3D-GAF) model allows for the inclusion of anisotropic collective motions into the analysis[17,18]. In the current investigation, this is applied to the YadAM β-barrel.

In the SMF model, the motions were modeled as isotropic motions. For the 3D-GAF model, we analyze the data by adopting the model from Lewandowski et al.[18], Saurel et al.[19] and Good et al.[23] who presented a modified SMF model in which motions are considered as *anisotropic* collective motions. The order parameter S^2 for the isotropic motions was replaced with an anisotropic order parameter $S^2_{\mu\nu}$, which parametrizes the motion as Gaussian fluctuations against three orthogonal axes, α, β and γ. As experimental data we used only ^{15}N R_1 and $R_{1\rho}$ rates as input to the 3D-GAF model as the model requires the specification of the interaction axis that can only be done for N-H bond vectors.

The spectral density for 3D-GAF is given as

$$J(\omega) = (1 - S^2_{s,\mu\nu})\,\frac{\tau_{s,\mu\nu}}{1+(\omega\tau_{s,\mu\nu})^2} \tag{25}$$

where $(\tau_{s,\mu\nu}, S^2_{s,\mu\nu})$ are the order parameter and correlation time for the anisotropic collective motion.

$S^2_{s,\mu\nu} =$

$$\frac{4\pi}{5}\sum_{l,k,k',m,m'}^{2}(-i)^{k-k'}e^{-\frac{\sigma_\alpha^2}{l^2}-\frac{\sigma_\beta^2\left(k^2+k'^2\right)}{2}-\frac{\sigma_\gamma^2\left(m^2+m'^2\right)}{2}}d_{kl}^{(2)}\left(\frac{\pi}{2}\right)d_{k'l}^{2}\left(\frac{\pi}{2}\right)d_{m'k'}^{2}\left(\frac{\pi}{2}\right)Y_{2m}\left(e_\mu\right)Y_{2m'}^{*}(e_\nu) \quad (26)$$

Here, σ_α, σ_β and σ_γ are the amplitudes of the Gaussian fluctuations against the orthogonal axes of motion (α, β and γ). $d_{kl}^{(2)}\left(\frac{\pi}{2}\right)$ are the reduced Wigner rotation matrix elements and Y_{2m} the second-order spherical harmonics as function of the angular coordinates $e_\mu = (\theta_\mu, \varphi_\mu)$ and $e_\nu = (\theta_\nu, \varphi_\nu)$ in the 3D GAF reference frame. μ and ν are the interacting NH amide bond vectors whose fluctuations are responsible for the observed relaxation[14-17] .

If the fluidity in the membrane is high, axial diffusion of the protein along the transverse membrane axis is expected[19] whereas if the fluidity is low then rather a rocking motion is expected. A rocking motion could also be present in hydrated crystals[21]. Although rocking motions have been studied in lipid environments by other groups, there are no reports of rocking motion of proteins in membrane vesicles. Therefore, it is interesting to study and compare anisotropic motions for the two types of preparations used in this study, membrane-embedded and microcrystalline YadAM, with the help of the 3D-GAF formalism.

In this approach, the amide NH bonds fluctuate around the three axes of the inertia of the protein. The corresponding order parameters hence depend on the angle between each amide bond vector and the molecular frame, and on the fluctuation amplitudes (σ_α, σ_β, σ_γ) of the collective motions. The rates are the averaged dynamics of the three monomers. Considering the rotations around the three inertia axes of the protein trimer and assuming that YadAM trimers have cylindrical symmetry, the number of fit parameters can be reduced ($\sigma_\alpha = \sigma_{||}, \sigma_\beta = \sigma_\gamma = \sigma_\perp$).

The fit parameters were determined by minimizing χ^2 function for trimeric protein.

$$I_{Rm}^{\mathrm{calc}}(t_{\mathrm{k}}) = I_{Rm}\exp\left(-R_m\left(S_{s,\mu\nu}^2, \tau_{s,\mu\nu}, \omega\right)t_{\mathrm{k}}\right) \quad (27)$$

$$\chi^2 = \left[\frac{1}{N}\sum_{k=1}^{N}\left(\frac{I_{15\mathrm{NR1}}^{\mathrm{expt}}(t_k)-I_{15\mathrm{NR1}}^{\mathrm{calc}}(t_k)}{\sigma_{15\mathrm{NR1,expt}}^2}\right)^2 + \frac{1}{N}\sum_{k=1}^{N}\left(\frac{I_{15\mathrm{NR1\rho}}^{\mathrm{expt}}(t_k)-I_{15\mathrm{NR1\rho}}^{\mathrm{calc}}(\mathrm{t_k})}{\sigma_{15\mathrm{NR1\rho,expt}}^2}\right)^2\right] \quad (28)$$

where i is the residue number and N is number of experimental relaxation data points.

We observed elevated rates in the loop regions, β2 and β3, most pronounced in the G74 to A82 region, indicating significant internal motions. To capture the global motion precisely, residues with significant internal motions were excluded from the SMF-3D GAF model fitting[16]. The following residues were included for the analysis of YadAM-Mx: S44, Y49, N55, F56, A58, A70, G72, S73, G86, V87, 88A, 89Y, M96, A99, S100, F101. For YadAM-OM, the flowing residues were included for the analysis: N43, A58, G72, G86, V87, A88, S100, F101. With this model, the angular fluctuations amplitudes $\sigma_{||}$ and $\sigma_\perp$ were determined as 6.4 ± 0.24°, and 6.15 ± 0.07° and for YadAM-Mx, respectively, and as 6.35 ± 0.14° and 8.36 ± 0.4° for YadAM-OM. The correlation time $\tau_{s,\mu\nu}$ was 41.5 ± 1.1 ns for YadAM-Mx and 40.8 ± 0.6 ns for YadAM-OM.

Text S8: Grid search analysis for 3D GAF model

In order to analyze if there exists an extended range of motional amplitudes that can describe the experimental data equally well, we performed a grid search on the amplitudes ($\sigma_{||}, \sigma_{\perp}$). This grid search was performed by stepwise fixing both σ values individually in the range from 1° to 15° in steps of 0.5°. For each pair of fixed amplitudes, we optimized the remaining fit parameters which are $\tau_{s,\mu\nu}$, I_{Rm} , ^{15}N R_1 and ^{15}N $R_{1\rho}$. The best fit parameters were estimated by minimizing the χ^2 function (Eq.28).

Based on the grid search, we found optimal ($\sigma_{||}, \sigma_{\perp}$) values around (6.5°, 6°) for YadAM-Mx and (6.5°, 8.5°) for YadAM-OM. Based on these values we could calculate a $\tau_{\mathrm{s,col}}$ of 41.5 ± 1.1 ns for YadAM-Mx and of 40.8 ± 2 ns for YadAM-OM. From the grid search it transpires that for YadAM in microcrystals the fluctuation amplitudes $(\sigma_{||}, \sigma_{\perp})$ are quite symmetric. However, for YadAM in the OM we observed a larger angular fluctuation amplitude around the $\sigma_{||}$ axis as compared to the $\sigma_{\perp}$ axis.

Figures

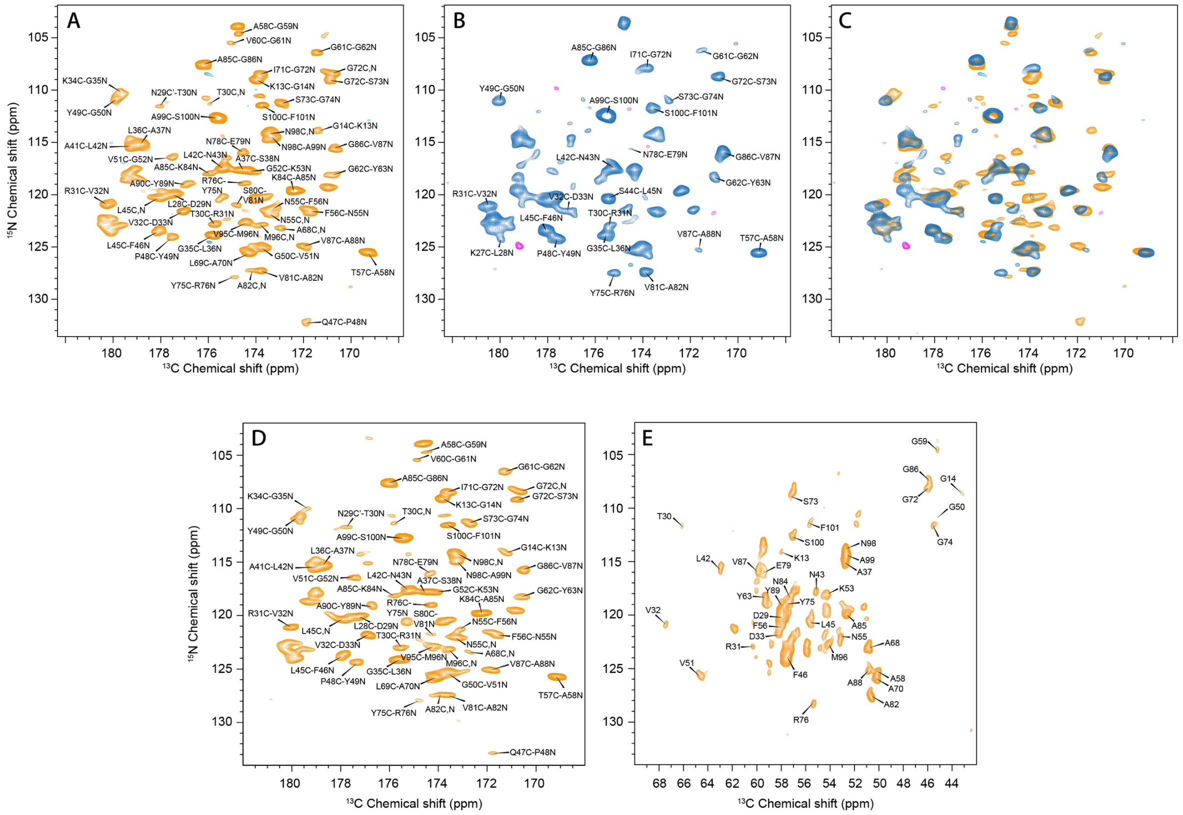

Figure S1. 2D NCO and NCA spectra of YadAM. The NCO spectra of YadAM-Mx (**A**) and YadAM-OM (**B**) are displayed with assignments. In **B** those peaks were labelled that were included into the analysis. (**C**) Overlay of the 2D NCO spectra of YadAM-Mx (orange) and YadAM-OM (blue). Side-by-side comparison of NCO (**D**) and NCA (**E**) spectra of YadAM-Mx, displayed with assignments.

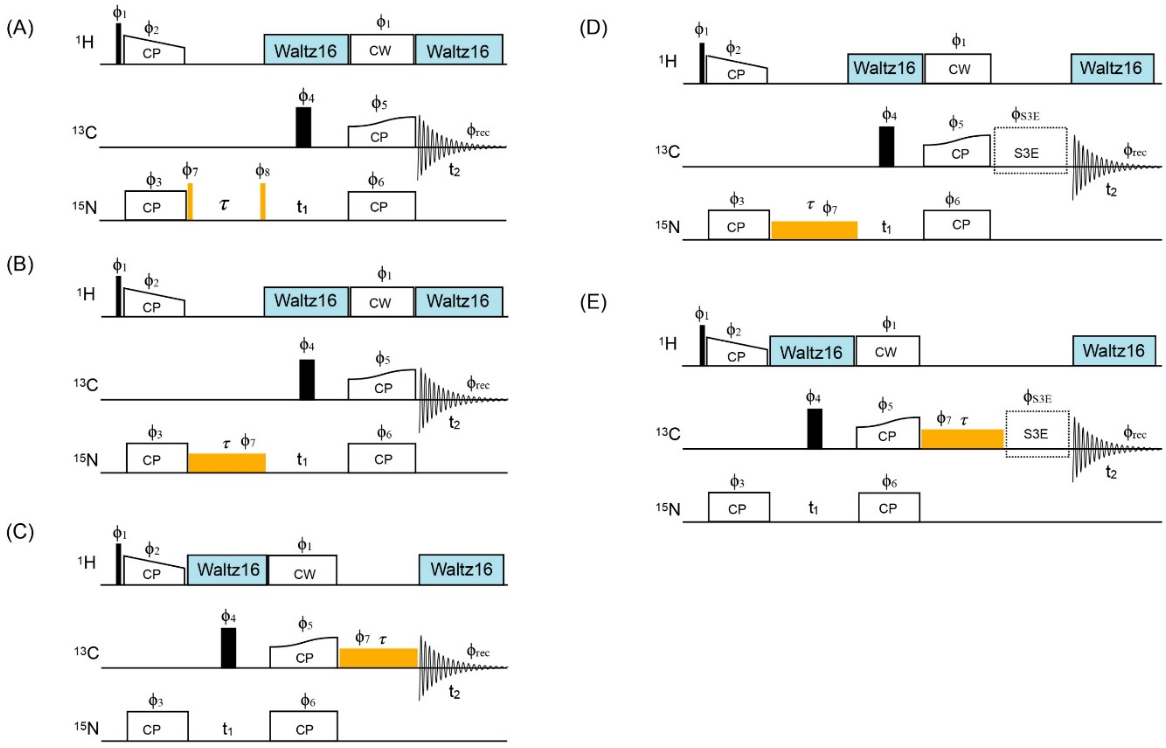

Figure S2. Pulse sequences used for the relaxation measurements. The 2D ^{15}N-^{13}C' DCP pulse sequences for ^{15}N R_1 (A), ^{15}N $R_{1\rho}$ (B), and ^{13}C' $R_{1\rho}$ (C) measurements were employed for investigating YadAM-OM relaxation. YadAM-Mx relaxation was investigated using the 2D ^{15}N-^{13}C' DCP sequence in (A) for ^{15}N R_1, the 2D ^{15}N-^{13}C' DCP-S3E pulse sequence for ^{15}N $R_{1\rho}$ (D) and ^{13}C' $R_{1\rho}$ (E). All sequences were run as pseudo 3D experiments. The DCP-S3E data were processed using the "splitcomb" command in Topspin. For selective excitation of ^{13}C$_\alpha$ and ^{13}C' regions, the Gaussian Cascade pulse shape was used to selectively excite the C' region whereas Sinc-shaped pulses were used to selectively excite the C$_\alpha$ region. A Waltz16 heteronuclear decoupling sequence with an RF power of ~10 kHz was applied during t_1 and t_2 periods in all pulse sequences. During the second CP period, a CW pulse was applied on the ^{1}H channel.

Phase cycling used for the above pulse sequences:

(A) ϕ_1 = (+y, -y), ϕ_2 = ϕ_3 = ϕ_4 = (+x), ϕ_5 = (+x, +x, +x, +x, +y, +y, +y, +y, -x, -x, -x, -x, -y, -y, -y, -y), ϕ_6 = (+x, +x, +x, +x, -x, -x, -x, -x), ϕ_7 = (+y, +y, -y, -y), ϕ_8 = (-y, -y, y, y), ϕ_{rec} = (+y, -y, y, -y, +x, -x, +x, -x, -y, +y, -y, +y, -x, x, -x, x)

(B) ϕ_1 = (+y, -y), ϕ_2 = ϕ_3 = ϕ_4 = ϕ_7 = (+x), ϕ_5 = (+x, +x, +x, +x, +y, +y, +y, +y, -x, -x, -x, -x, -y, -y, -y, -y) ϕ_6 = (+x, +x, +x, +x, -x, -x, -x, -x), ϕ_{rec} = (y, -y, y, -y, x, -x, x, -x, -y, y, -y, y, -x, x, -x, x)

(C) ϕ_1 = (+y, +y, +y, +y, -y, -y, -y, -y), ϕ_2 = ϕ_4 = (+x), ϕ_3 = (+x, +x, +x, +x, +x, +x, +x, +x, -x, -x, -x, -x, -x, -x, -x, -x), ϕ_5 = (+x +x, -x, -x), ϕ_6 = (+x, -x, +x, -x), ϕ_{rec} = (+x, -x, -x, +x, -x, +x, +x, -x, -x, +x, +x, -x, +x, -x, -x, +x)

(D) ϕ_1 = (+y, -y), ϕ_2 =ϕ_3 =ϕ_4 = ϕ_6 = ϕ_{S3E} =(+x), ϕ_5=ϕ_7 = (+x, +x, -x, -x [A]/-y, -y, +y, +y[B]), ϕ_{rec} = (+x, -x, +x, -x)

(E) ϕ_1 = (+y, -y), ϕ_2 =ϕ_3 =ϕ_4 = ϕ_6 =ϕ_6 = ϕ_{S3E} =(+x), ϕ_5 = (+x, +x, -x, -x, [A]/-y, -y, +y, +y[B]), ϕ_{rec} = (+x, -x, +x, -x)

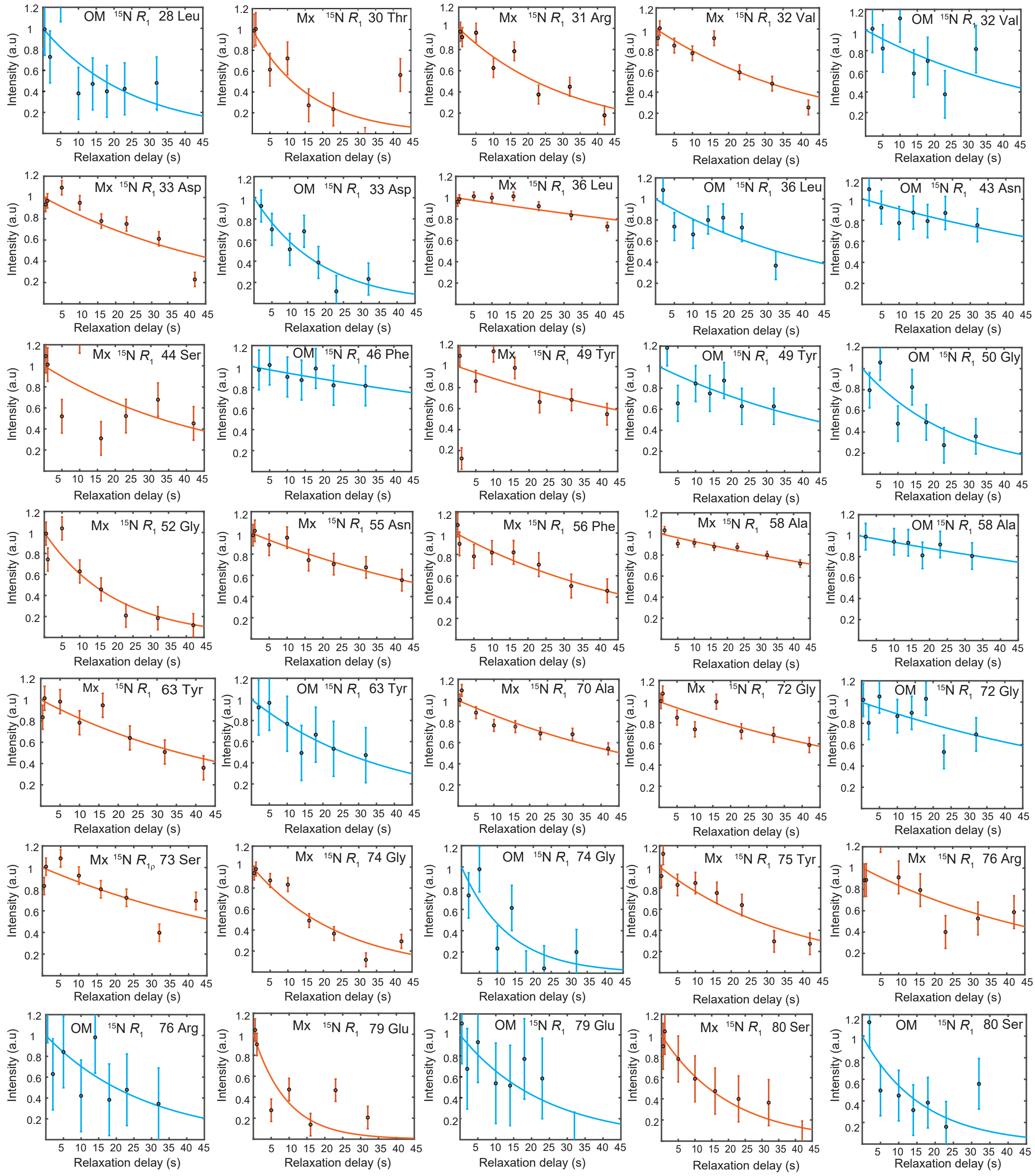

OM 15N R1 28 Leu
Mx 15N R1 30 Thr
Mx 15N R1 31 Arg
Mx 15N R1 32 Val
OM 15N R1 32 Val
Mx 15N R1 33 Asp
OM 15N R1 33 Asp
Mx 15N R1 36 Leu
OM 15N R1 36 Leu
OM 15N R1 43 Asn
Mx 15N R1 44 Ser
OM 15N R1 46 Phe
Mx 15N R1 49 Tyr
OM 15N R1 49 Tyr
OM 15N R1 50 Gly
Mx 15N R1 52 Gly
Mx 15N R1 55 Asn
Mx 15N R1 56 Phe
Mx 15N R1 58 Ala
OM 15N R1 58 Ala
Mx 15N R1 63 Tyr
OM 15N R1 63 Tyr
Mx 15N R1 70 Ala
Mx 15N R1 72 Gly
OM 15N R1 72 Gly
Mx 15N R1ρ 73 Ser
Mx 15N R1 74 Gly
OM 15N R1 74 Gly
Mx 15N R1 75 Tyr
Mx 15N R1 76 Arg
OM 15N R1 76 Arg
Mx 15N R1 79 Glu
OM 15N R1 79 Glu
Mx 15N R1 80 Ser
OM 15N R1 80 Ser
Intensity (a.u)
Relaxation delay (s)

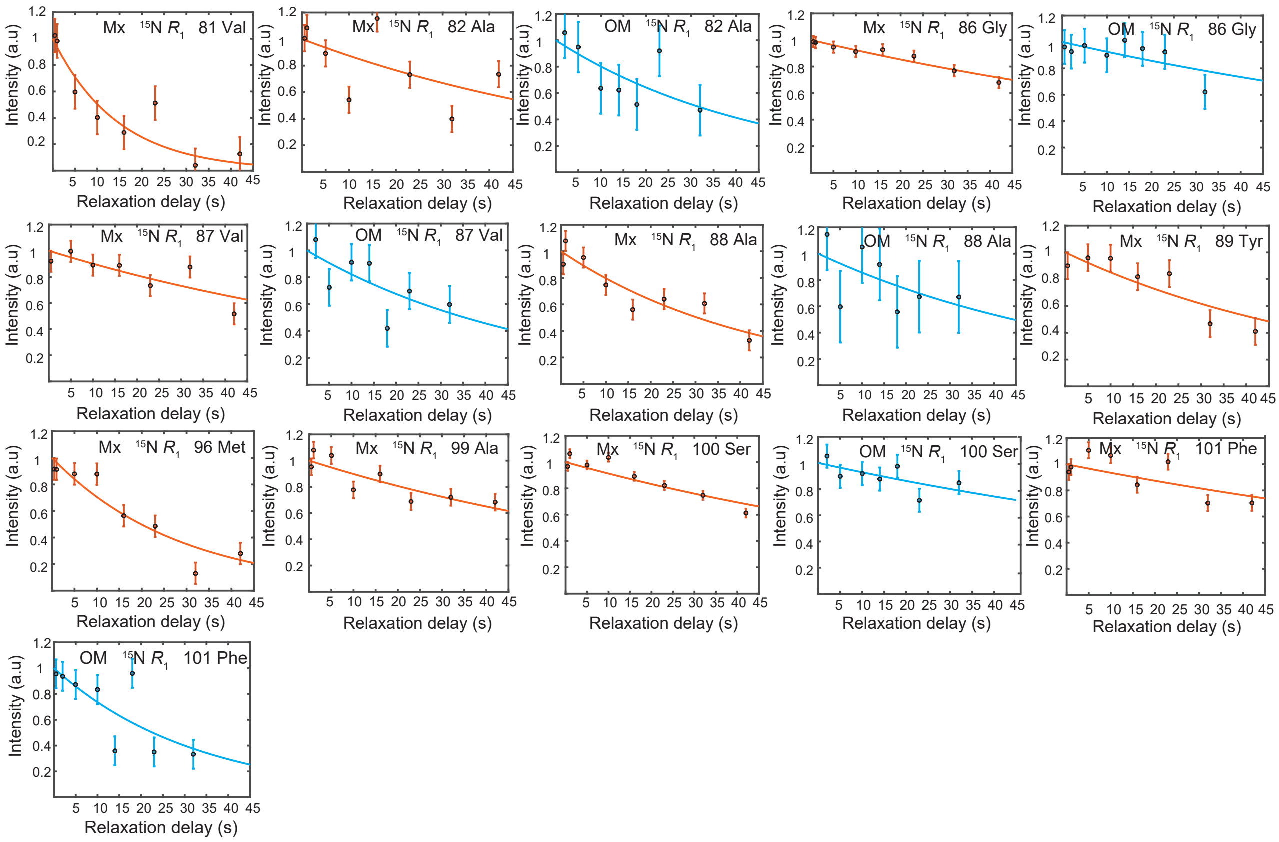

Figure S3. ^{15}N R_1 decay curves of YadAM-Mx (orange) and YadAM-OM (blue). Measurements were performed at a magnetic field strength of 21.1 T and a MAS frequency of 60 kHz. The sample temperature was set to 280 K for YadAM-Mx and 286 K for YadAM-OM.

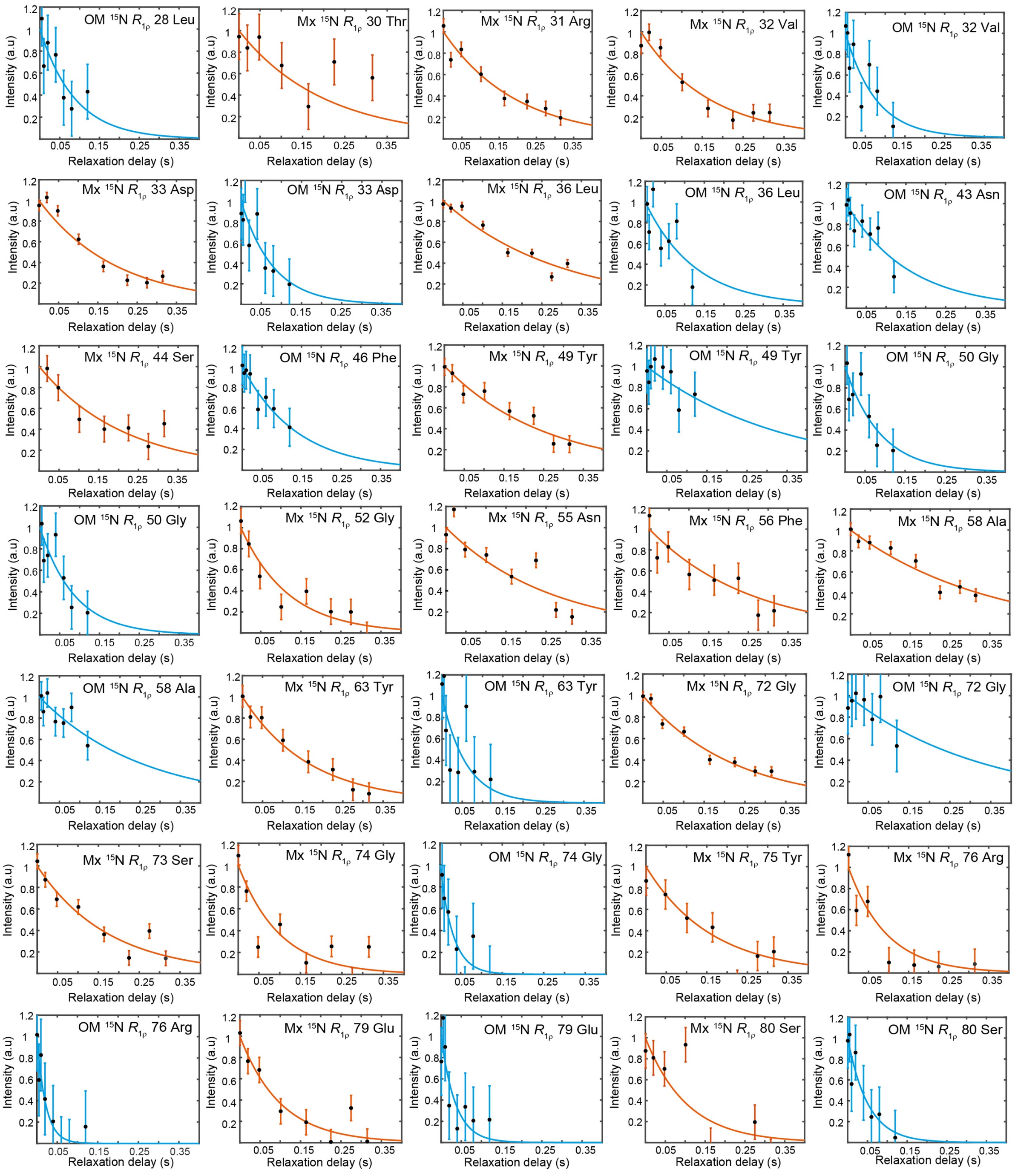

OM ^{15}N $R_{1\rho}$ 28 Leu
Mx ^{15}N $R_{1\rho}$ 30 Thr
Mx ^{15}N $R_{1\rho}$ 31 Arg
Mx ^{15}N $R_{1\rho}$ 32 Val
OM ^{15}N $R_{1\rho}$ 32 Val
Mx ^{15}N $R_{1\rho}$ 33 Asp
OM ^{15}N $R_{1\rho}$ 33 Asp
Mx ^{15}N $R_{1\rho}$ 36 Leu
OM ^{15}N $R_{1\rho}$ 36 Leu
OM ^{15}N $R_{1\rho}$ 43 Asn
Mx ^{15}N $R_{1\rho}$ 44 Ser
OM ^{15}N $R_{1\rho}$ 46 Phe
Mx ^{15}N $R_{1\rho}$ 49 Tyr
OM ^{15}N $R_{1\rho}$ 49 Tyr
OM ^{15}N $R_{1\rho}$ 50 Gly
OM ^{15}N $R_{1\rho}$ 50 Gly
Mx ^{15}N $R_{1\rho}$ 52 Gly
Mx ^{15}N $R_{1\rho}$ 55 Asn
Mx ^{15}N $R_{1\rho}$ 56 Phe
Mx ^{15}N $R_{1\rho}$ 58 Ala
OM ^{15}N $R_{1\rho}$ 58 Ala
Mx ^{15}N $R_{1\rho}$ 63 Tyr
OM ^{15}N $R_{1\rho}$ 63 Tyr
Mx ^{15}N $R_{1\rho}$ 72 Gly
OM ^{15}N $R_{1\rho}$ 72 Gly
Mx ^{15}N $R_{1\rho}$ 73 Ser
Mx ^{15}N $R_{1\rho}$ 74 Gly
OM ^{15}N $R_{1\rho}$ 74 Gly
Mx ^{15}N $R_{1\rho}$ 75 Tyr
Mx ^{15}N $R_{1\rho}$ 76 Arg
OM ^{15}N $R_{1\rho}$ 76 Arg
Mx ^{15}N $R_{1\rho}$ 79 Glu
OM ^{15}N $R_{1\rho}$ 79 Glu
Mx ^{15}N $R_{1\rho}$ 80 Ser
OM ^{15}N $R_{1\rho}$ 80 Ser
Intensity (a.u)
Relaxation delay (s)

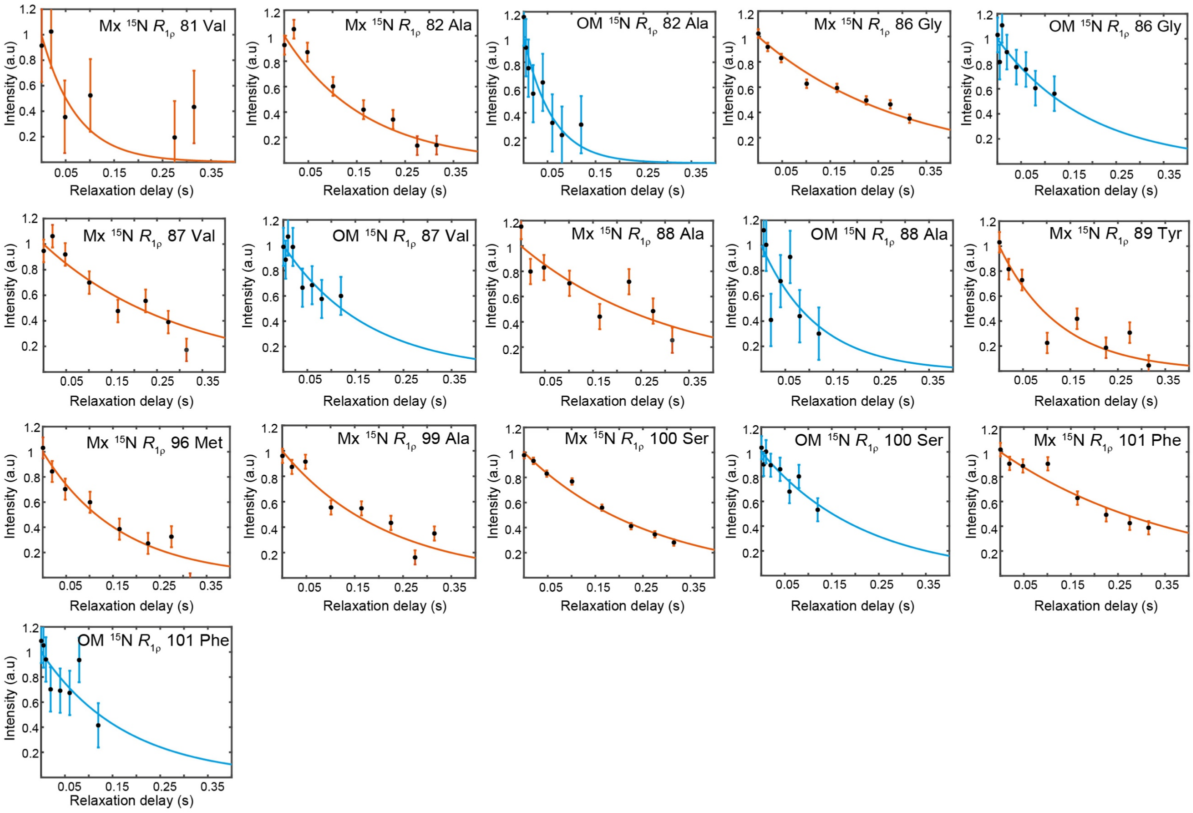

Figure S4. ^{15}**N** $\boldsymbol{R_{1\rho}}$ **decay curves of YadAM-Mx (orange) and YadAM-OM (blue).** Measurements were performed at a magnetic field strength of 21.1 T and a MAS frequency of 60 kHz. The sample temperature was set to 280 K for YadAM-Mx and 286 K for YadAM-OM.

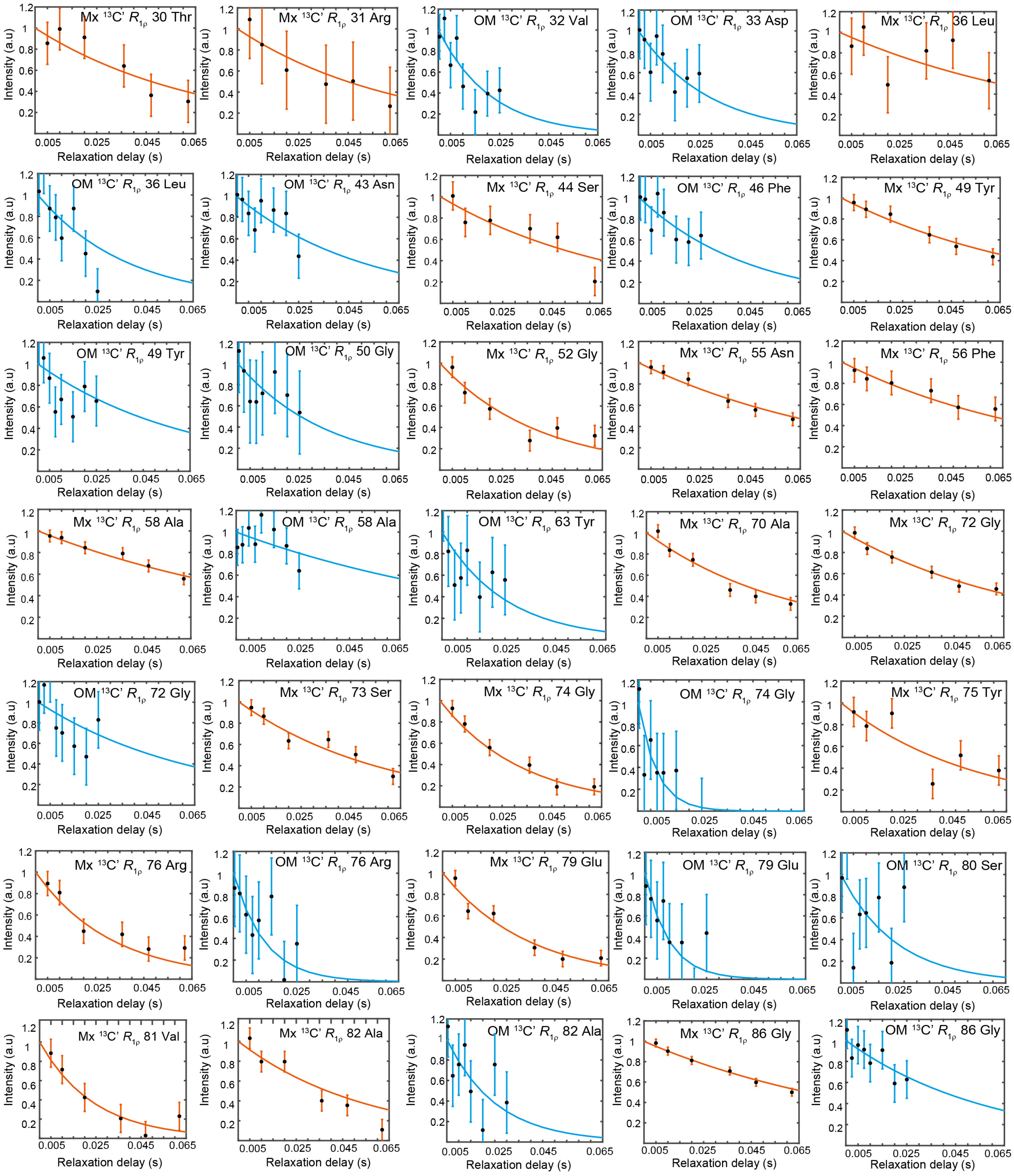
Mx 13C' R1ρ 30 Thr
Mx 13C' R1ρ 31 Arg
OM 13C' R1ρ 32 Val
OM 13C' R1ρ 33 Asp
Mx 13C' R1ρ 36 Leu
OM 13C' R1ρ 36 Leu
OM 13C' R1ρ 43 Asn
Mx 13C' R1ρ 44 Ser
OM 13C' R1ρ 46 Phe
Mx 13C' R1ρ 49 Tyr
OM 13C' R1ρ 49 Tyr
OM 13C' R1ρ 50 Gly
Mx 13C' R1ρ 52 Gly
Mx 13C' R1ρ 55 Asn
Mx 13C' R1ρ 56 Phe
Mx 13C' R1ρ 58 Ala
OM 13C' R1ρ 58 Ala
OM 13C' R1ρ 63 Tyr
Mx 13C' R1ρ 70 Ala
Mx 13C' R1ρ 72 Gly
OM 13C' R1ρ 72 Gly
Mx 13C' R1ρ 73 Ser
Mx 13C' R1ρ 74 Gly
OM 13C' R1ρ 74 Gly
Mx 13C' R1ρ 75 Tyr
Mx 13C' R1ρ 76 Arg
OM 13C' R1ρ 76 Arg
Mx 13C' R1ρ 79 Glu
OM 13C' R1ρ 79 Glu
OM 13C' R1ρ 80 Ser
Mx 13C' R1ρ 81 Val
Mx 13C' R1ρ 82 Ala
OM 13C' R1ρ 82 Ala
Mx 13C' R1ρ 86 Gly
OM 13C' R1ρ 86 Gly
Intensity (a.u)
Relaxation delay (s)

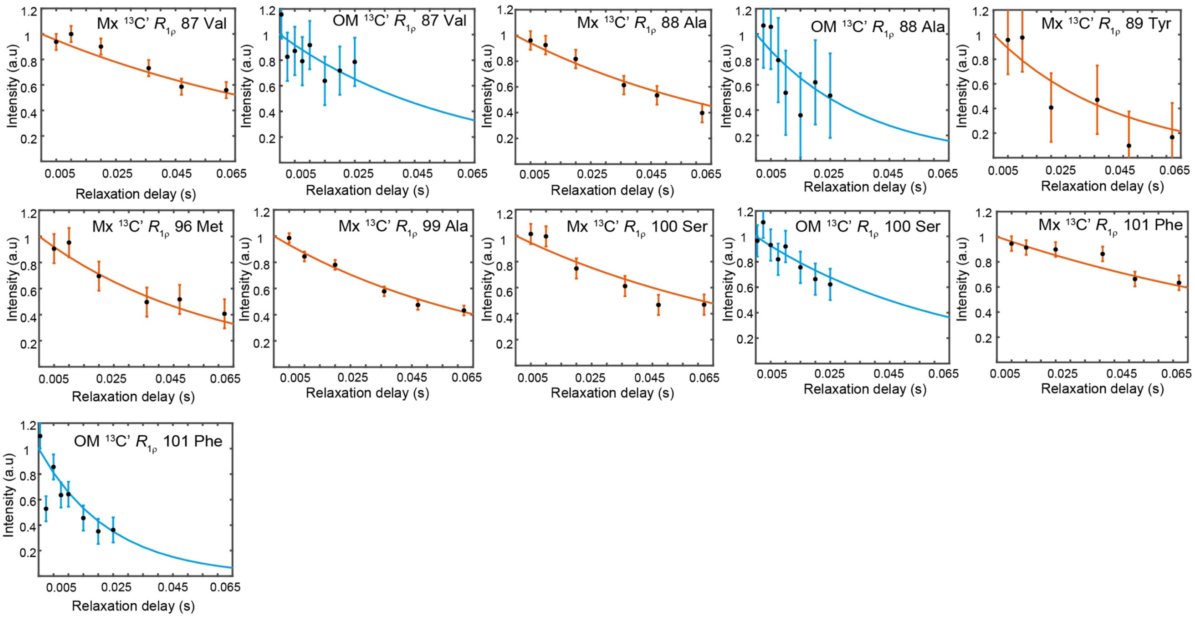

Figure S5. ^{13}C’ $R_{1\rho}$ decay curves of YadAM-Mx (orange) and YadAM-OM (blue). Measurements were performed at a magnetic field strength of 21.1 T and a MAS frequency of 60 kHz. The sample temperature was set to 280 K for YadAM-Mx and 286 K for YadAM-OM.

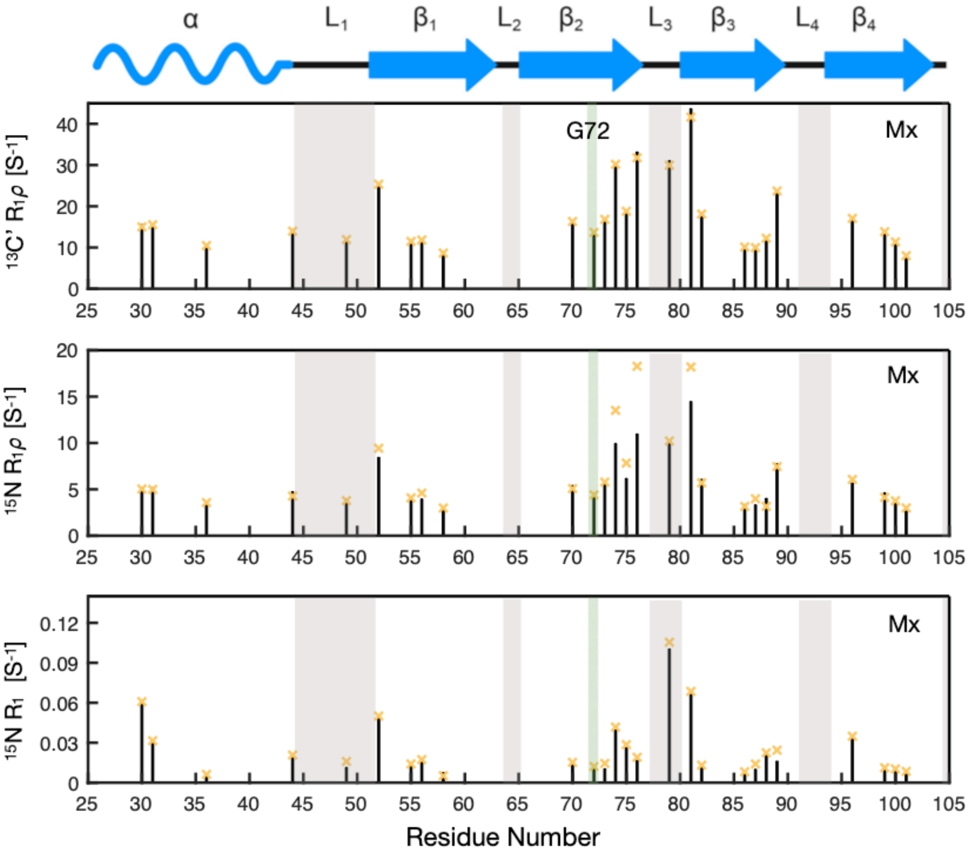

Figure S6. Relaxation rates back-calculated from the SMF model for YadAM-Mx (text S5). The symbol x (orange) denotes the experimental rates, while vertical bars (black) represent the back-calculated rates. The rates were back calculated with fixed fit parameters.

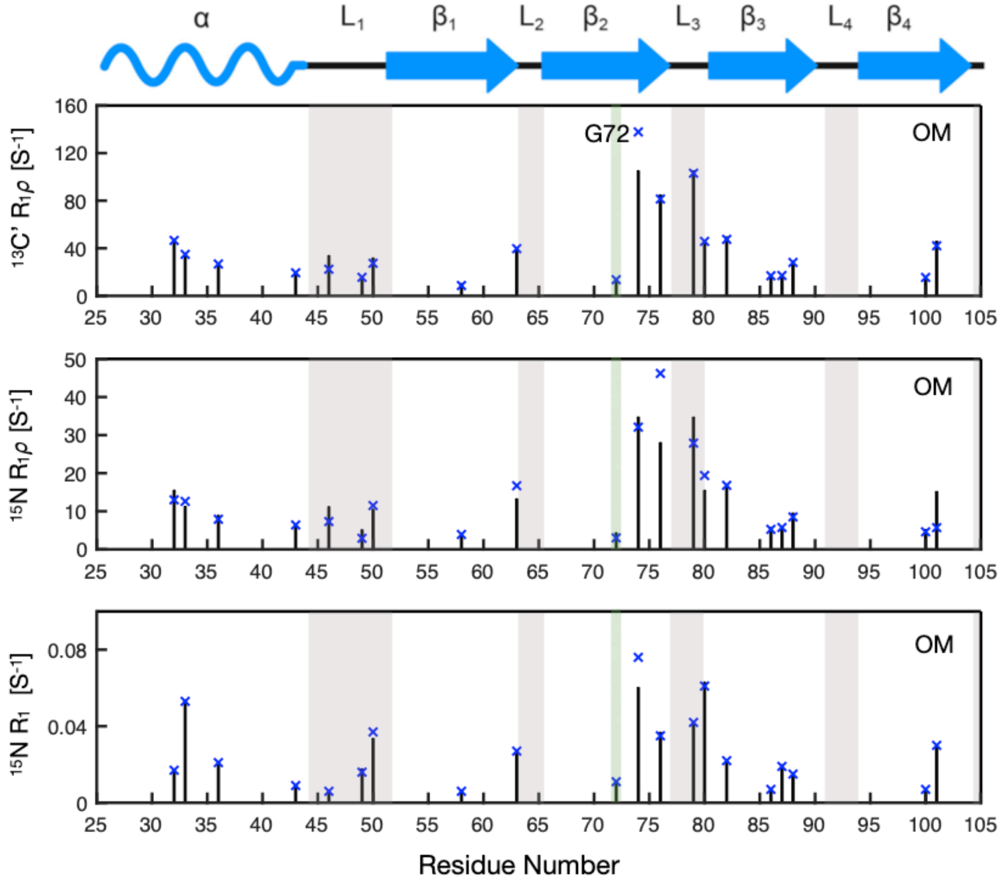

Figure S7. Relaxation rates back-calculated from the SMF model for YadAM-OM (text S5). The symbol x (blue) denotes the experimental rates, while vertical bars (black) represent the back-calculated rates. The rates were back calculated with fixed fit parameters.

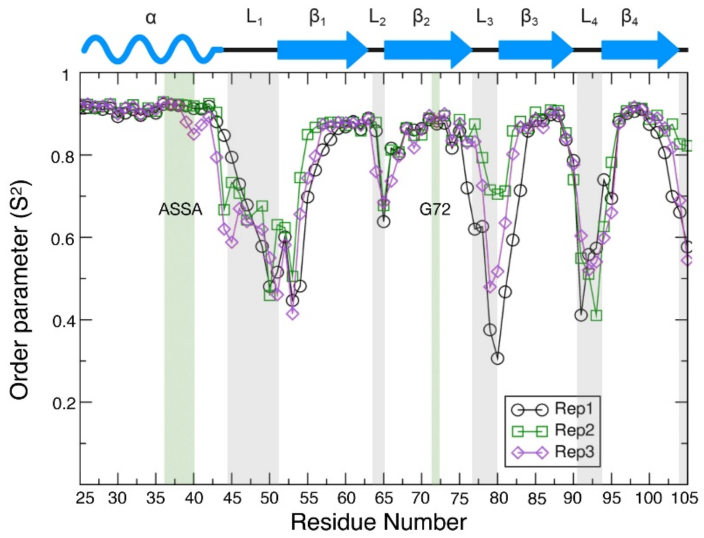

Figure S8. **MD-derived order parameters from three 2-μs simulation replicas (YadAM-OM).** The three replicas are shown as black, green and red lines.

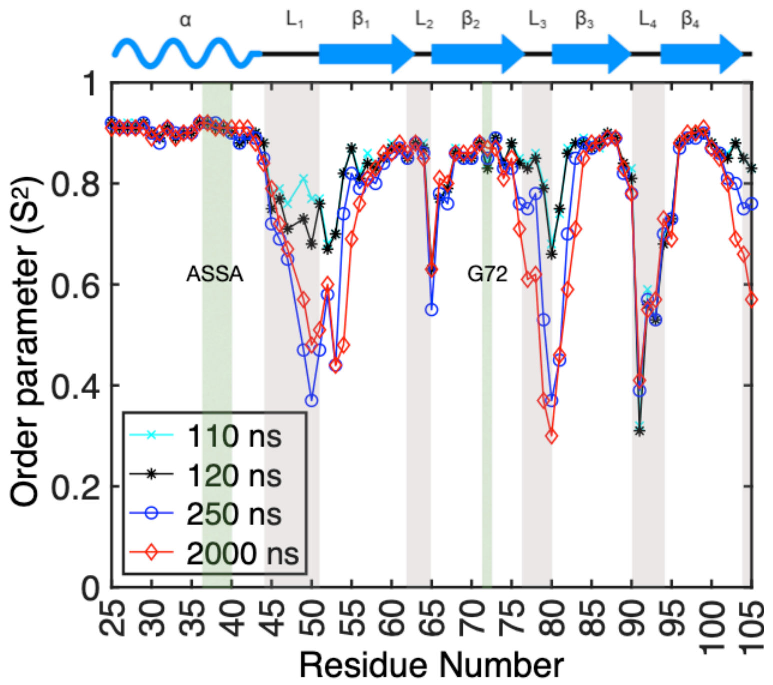

Figure S9. MD-derived order parameters of YadAM-OM. They were obtained by analysing different portions of the 2-μs simulations, 110 ns (cyan), 120 ns (black), 250 ns (blue) and 2000 ns (red). The order parameters are averaged over three replicas.

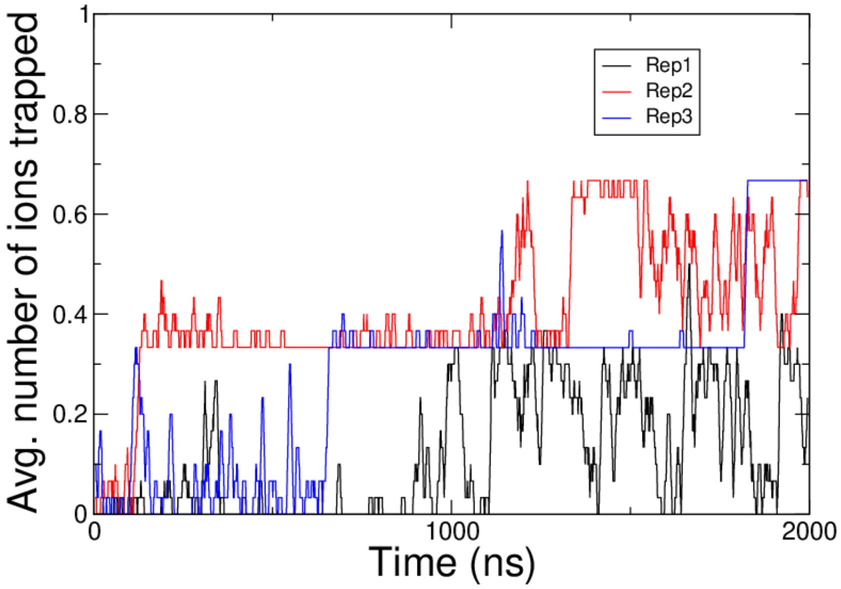

Figure S10. Calcium ions trapped in MD simulations for YadAM-OM. The graph shows the average number per monomer of calcium ions trapped in the highly conserved region (75-80), over the 2-μs range of the MD simulation. The data are shown for the three replicas.

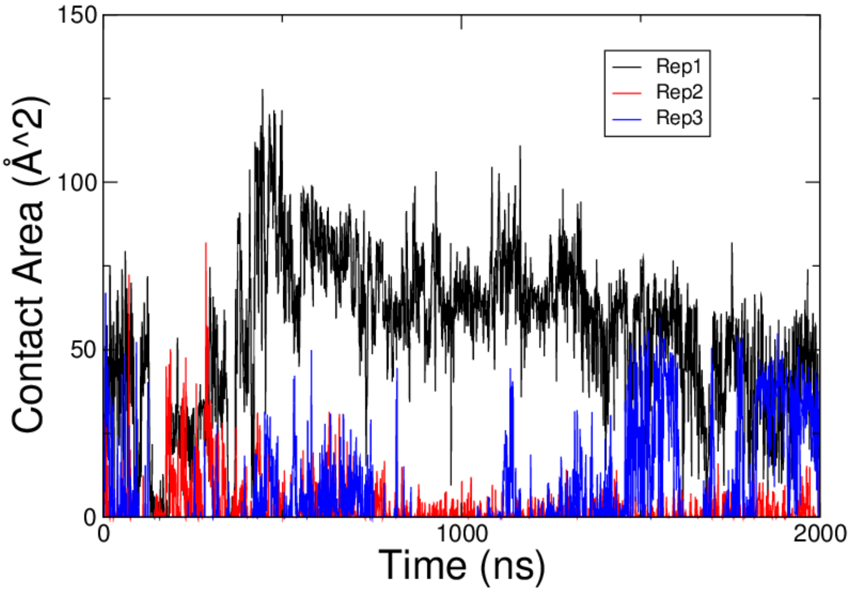

Figure S11. **Contact area between residues Y49 and E79 in simulations of YadAM-OM.** The three 2-μs simulation replicas are shown as black, red, and green lines.

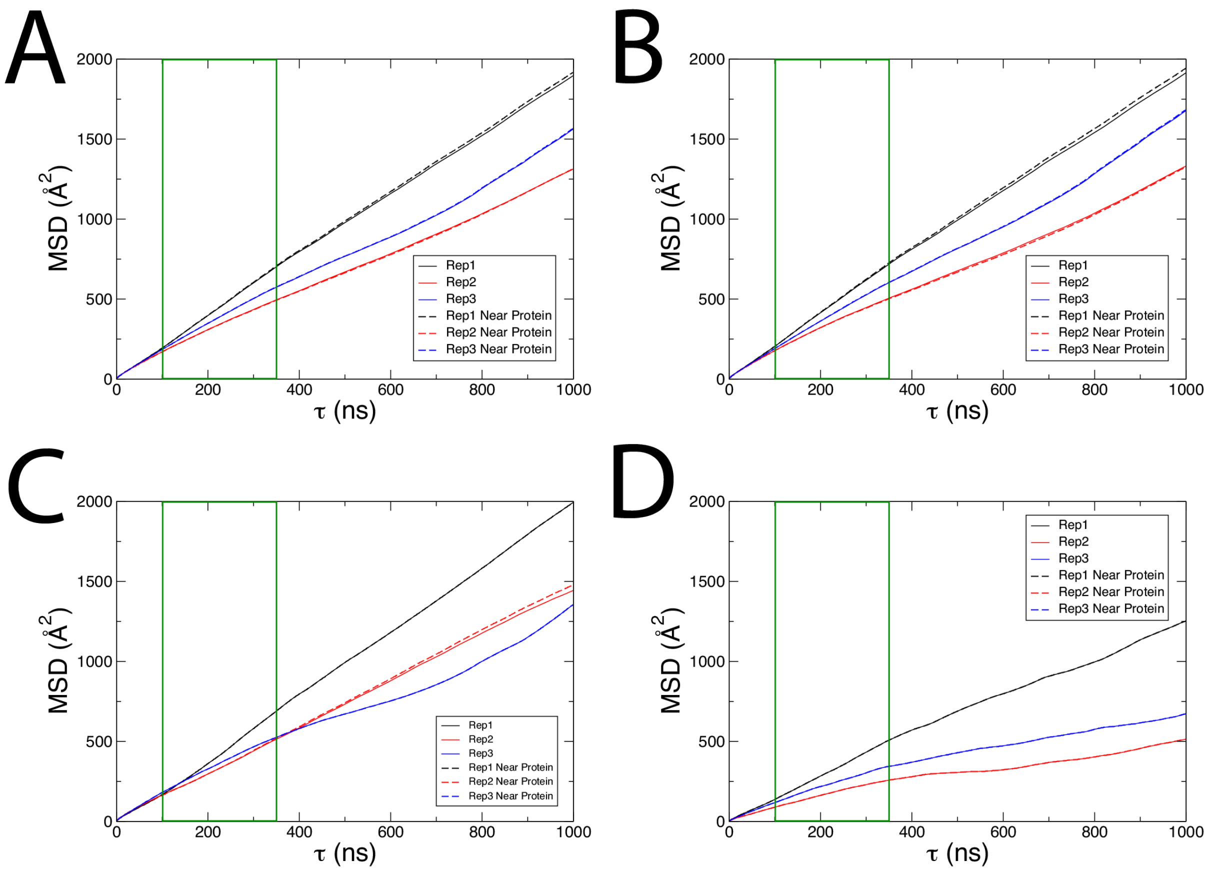

Figure S12. Mean squared displacement (MSD) different lipids over different time intervals. The panels show MSD over time for all periplasmic lipids (A), PPPE (B), PVPG (C), and PVCL2 (D). Each replica is shown in black, red, and blue, respectively. The MSD over time for the lipids near the protein are shown in dashed lines.

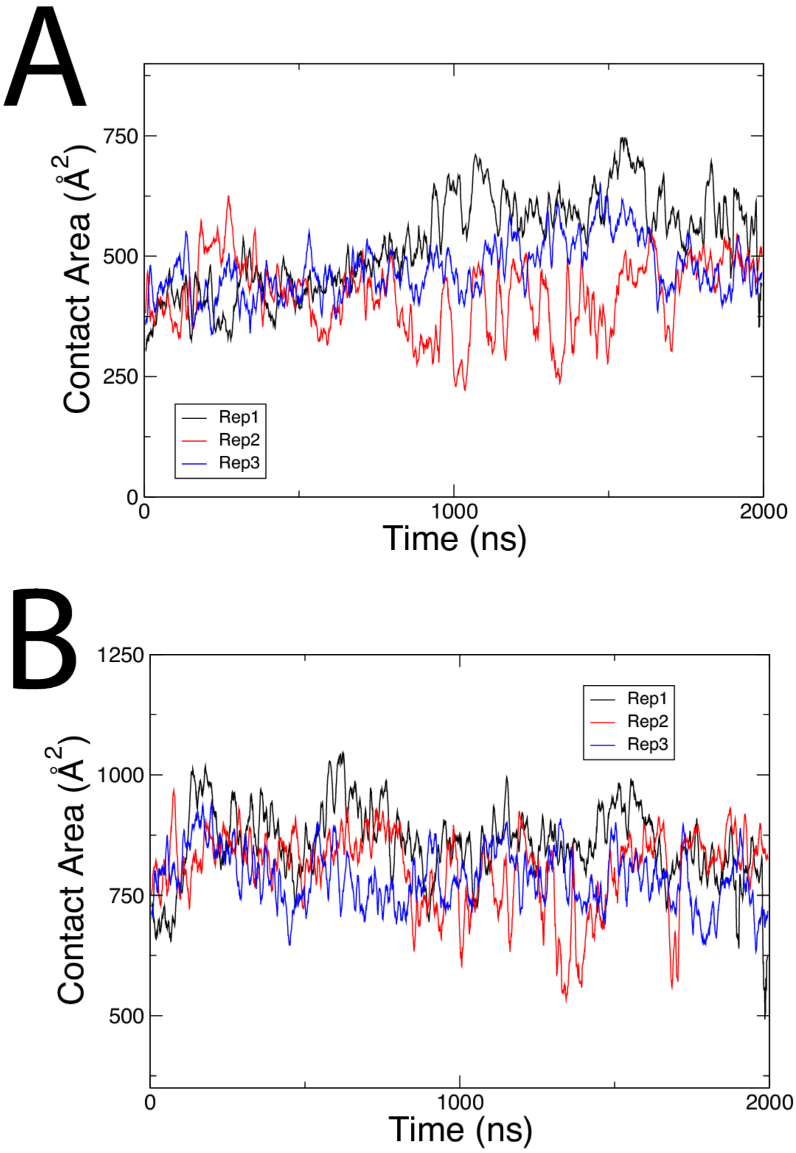

Figure S13. Contact between periplasmic loops and lipids over time. Panel A shows contact made between the first periplasmic loop (residues 49 to 54) and panel B the second periplasmic loop (residues 75 to 80). The data shown are for all three replicas.

Table S1. Experimental parameters used to acquire the NMR data.

	^{1}H	^{13}C	^{15}N
90°	100 kHz	50	35.7
$R_{1\rho}$ (spin-lock)	17 kHz	17 kHz	17 kHz
Waltz16 (decoupling)	~12 kHz	----	----
First CP	Ramp.80-100	----	Square.100
rf on first CP	~ 48.3 kHz (YadAM-Mx)<br>~ 51.5 kHz (YadAM-OM)	----	~13.68 kHz (YadAM-Mx)<br>~11.12 kHz (YadAM-OM)
1st CP contact time	2 ms (YadAM-Mx)<br>1 ms (YadAM-OM)	----	2 ms (YadAM-Mx)<br>1 ms (YadAM-OM)
Second CP	CW	Tangential pulse	Square.100
rf on second CP	15.5 kHz (YadAM-Mx)<br>12.8 kHz (YadAM-OM)	~17.5 kHz (YadAM-Mx)<br>~39.1 kHz (YadAM-OM)	~45.8 kHz (YadAM-Mx)<br>~32.6 kHz (YadAM-OM)
2nd CP contact time	---	9 ms (YadAM-Mx)<br>5 ms (YadAM-OM)	9 ms (YadAM-Mx)<br>5 ms (YadAM-OM)
Selective 180° C_{α} pulse	----	Sinc pulses (duration 450 μs)	----
Selective 180° C’ pulse	----	Gaussian cascade (duration 700 μs)	-----
t_2 acquisition	----	23 ms (YadAM-Mx)<br>17 ms (YadAM-OM)	----

Table S2. ^{15}N R_1, ^{15}N $R_{1\rho}$, and ^{13}C' $R_{1\rho}$ relaxation rates of YadAM-Mx and YadAM-OM.

Residue	YadAM–Mx			YadAM–OM		
	^{15}N R_1 (s^{-1})	^{15}N $R_{1\rho}$ (s^{-1})	^{13}C' $R_{1\rho}$ (s^{-1})	^{15}N R_1 (s^{-1})	^{15}N $R_{1\rho}$ (s^{-1})	^{13}C' $R_{1\rho}$ (s^{-1})
28 Leu	----	----	----	0.039±0.010	11.3±3.0	----
30 Thr	0.060±0.020	5.1±1.7	15.1±3.5	----	----	----
31 Arg	0.031±0.004	4.9±0.5	15.5±1.4	----	----	----
32 Val	0.023±0.002	5.9±0.7	----	0.017±0.003	13.0±1.3	46.6±6.8
33 Asp	0.021±0.002	5.6±0.4	15.4±0.9	0.053±0.007	12.6±2.5	34.9±5.8
36 Leu	0.006±0.001	3.5±0.2	10.4±0.5	0.021±0.003	7.9±1.3	26.8±3.3
43 Asn	----	----	-----	0.009±0.001	6.4±0.5	19.4±2.5
44 Ser	0.020±0.007	4.2±1.2	13.9±2.4	----	----	----
46 Phe	----	----	----	0.006±0.002	7.3±0.6	22.4±3.8
49 Tyr	0.019±0.002	3.7±0.5	11.9±1.2	0.016±0.004	2.9±1.1	15.6±3.0
50 Gly	----	----	----	0.037±0.006	11.5±2.0	27.4±4.4
52 Gly	0.050±0.008	9.4±2.9	25.3±3.5	----	----	----
55 Asn	0.014±0.003	4.1±0.4	11.4±0.7	----	----	-----
56 Phe	0.017±0.004	4.6±1.1	11.8±1.6	----	----	----
58 Ala	0.005±0.001	2.9±0.2	8.6±0.7	0.006±0.001	3.9±0.4	8.7±1.6
63 Tyr	0.019±0.004	6.0±0.9	----	0.027±0.009	17±12	40±11
70 Ala	0.015±0.002	5.1±0.2	16.3±0.8	----	----	----
72 Gly	0.012±0.002	4.4±0.3	13.6±0.7	0.011±0.001	3.0±0.6	13.6±3.5
73 Ser	0.014±0.002	5.7±0.6	16.8±1.2	---	---	----
74 Gly	0.041±0.004	13.5±5.4	30.1±2.9	0.076±0.013	32.1±5.8	137±26
75 Tyr	0.028±0.004	7.8±1.2	18.8±2.3	----	----	----
76 Arg	0.019±0.005	18.2±6.1	31.7±3.9	0.035±0.011	46±13	81±17
79 Glu	0.105±0.045	10.2±2.6	29.9±2.8	0.042±0.011	28±10	103±30
80 Ser	0.047±0.017	9.6±1.6	----	0.061±0.025	19.4 ±4.0	45.8±8.5
81 Val	0.068±0.020	18.1±7.1	41.6±7.9	----	----	----
82 Ala	0.013±0.003	5.7±0.5	18.1±1.5	0.022±0.006	16.8±4.1	47.5±8.6
86 Gly	0.008±0.001	3.1±0.1	10.1±0.4	0.007±0.002	5.25±1.0	16.9±2.4
87 Val	0.014±0.002	3.9±0.5	9.9±0.8	0.019±0.004	5.73±1.0	17.1±2.8
88 Ala	0.022±0.003	3.1±0.5	12.2±1.2	0.015±0.003	8.5±0.9	28.1±2.7
89 Tyr	0.024±0.003	7.4±1.3	23.7±3.8	----	----	----
96 Met	0.034±0.004	6.1±0.7	17.1±1.6	----	----	----
99 Ala	0.011±0.001	4.1±0.3	13.8±0.8	----	----	----
100 Ser	0.010±0.001	3.7±0.1	11.3±0.4	0.007±0.001	4.6±0.3	15.5±0.7
101 Phe	0.008±0.001	2.9±0.2	8.1±0.6	0.030±0.006	5.7±1.1	42.1±2.6

Table S3. Fit parameters for SMF model applied to YadAM-Mx and YadAM-OM.

SMF analysis	YadAM-Mx			YadAM-OM		
Residue	S^2	τ_{eff} [ns]	χ^2	S^2	τ_{eff}[ns]	χ^2
28 Leu	----	----	----	---	---	---
30 Thr	0.80±0.04	20.1±3.9	4.2	---	-----	----
31 Arg	0.86±0.01	28.2±2.6	2.4	----	----	----
32 Val	---	---	---	0.82±0.02	64.7±7.6	1.9
33 Asp	---	--	---	0.71±0.02	31.4±1.7	1.4
36 Leu	0.95±0.01	56.1±4.7	5.6	0.83±0.01	44.7±2.4	3.3
43 Asn	----	----	----	0.91±0.01	57±6.5	1.1
44 Ser	0.89±0.01	32.3±6.2	4.4	----	----	----
46 Phe	---	---	---	0.91±0.02	94±29	1.7
49 Tyr	0.93±0.01	40.1±4.6	11.3	0.9±0.01	36.8±4.6	1.8
50 Gly	-----	----	----	0.79±0.02	38.3±4.4	1.7
52 Gly	0.77±0.02	28.4±2.9	3.0	----	----	----
55 Asn	0.92±0.01	36.9±5.5	5.2	----	----	----
56 Phe	0.91±0.01	32.1±4.1	1.3	----	----	----
58 Ala	0.95±0.01	41.8±2.7	2.1	0.95±0.01	51±7.1	1.3
63 Tyr	---	---	---	0.79±0.05	49±13	1.2
70 Ala	0.90±0.01	40.9±3.1	4.4	----	----	----
72 Gly	0.92±0.01	42.1±4.5	3.4	0.92±0.01	43±10	1.6
73 Ser	0.92±0.01	50.1±5.5	5.4	----	----	----
74 Gly	0.77±0.02	34±2	5.9	0.3±0.02	52.0 ±7.6	2.0
75 Tyr	0.86±0.01	33.2±3.3	5.2	----	----	----
76 Arg	0.85±0.02	55±15	3.9	0.6±0.1	60 ±11	1.1
79 Glu	0.60±0.1	21.9±3.3	6.8	0.5±0.1	63 ±16	1.0
80 Ser	---	---	---	0.61±0.1	33.9 ±6.7	2.5
81 Val	0.62±0.1	31.9±4.8	3.1	----	----	----
82 Ala	0.90±0.01	47.2±7.4	7.1	0.79±0.04	60 ±11	1.6
86 Gly	0.94±0.01	45.3±3.9	2.3	0.93±0.01	60 ±12	1.1
87 Val	0.94±0.01	39.2±4.0	4.8	0.88±0.02	36.6 ±4.4	1.9
88 Ala	0.90±0.01	30.0±2.5	3.7	0.87±0.02	54.0±7.5	1.8
89 Tyr	0.88±0.01	47.5±5.1	6.8	----	----	----
96 Met	0.85±0.01	28.1±2.2	3.1	----	----	----
99 Ala	0.92±0.01	45.1±4.1	4.7	----	----	----
100 Ser	0.94±0.01	43.9±1.9	5.9	0.93±0.01	58.2±7.9	1.5
101 Phe	0.95±0.01	43.7±4.7	5.0	0.75±0.02	48.6±2.7	28.4

Table S4. **Back-calculated rates from SMF model for YadAM-Mx and YadAM-OM.**

Residue	YadAM–Mx			YadAM–OM		
	^{15}N R_1 (s^{-1})	^{15}N $R_{1\rho}$ (s^{-1})	^{13}C' $R_{1\rho}$ (s^{-1})	^{15}N R_1 (s^{-1})	^{15}N $R_{1\rho}$ (s^{-1})	^{13}C' $R_{1\rho}$ (s^{-1})
30 Thr	0.0592±0.019	5.2±1.1	15.5±3.3	----	----	----
31 Arg	0.0304±0.004	5.17±0.48	15.6±1.5	----	----	----
32 Val	----	----	----	0.0175±0.003	15.6±1.1	47.1±3.4
33 Asp	---	---	----	0.0536±0.004	11.3±0.9	34.1±2.8
36 Leu	0.0052±0.001	3.48±0.17	10.53±0.51	0.021±0.002	8.94±0.55	27.1±1.7
43 Asn	----	----	-----	0.010±0.002	6.46±0.45	19.5±1.4
44 Ser	0.0214±0.007	4.74±0.81	14.3±2.5	----	----	----
46 Phe	----	----	----	0.006±0.004	11.2±0.8	33.9±2.4
49 Tyr	0.0116±0.0024	3.98±0.39	12.1±1.2	0.0178±0.003	5.15±0.94	15.6±2.8
50 Gly	----	----	----	0.0337±0.004	10.5±1.8	31.9±5.4
52 Gly	0.049±0.008	8.5±1.2	25.5±3.5	----	----	----
55 Asn	0.0134±0.0034	3.89±0.26	11.7±0.79	----	----	-----
56 Phe	0.018±0.0039	3.96±0.57	11.9±1.7	----	----	----
58 Ala	0.0077±0.0008	2.86±0.21	8.65±0.63	0.0063±0.0014	3.48±0.29	10.5±0.89
63 Tyr	----	----	----	0.0258±0.0089	13.3±3.4	40±10
70 Ala	0.0152±0.0021	5.43±0.27	16.4±0.83	----	----	----
72 Gly	0.012±0.0023	4.54±0.26	13.73±0.79	0.0111±0.003	4.4±1.2	13.2±3.7
73 Ser	0.0105±0.002	5.63±0.42	17.1±1.3	---	---	----
74 Gly	0.0397±0.003	9.95±0.85	30.1±2.6	0.0603±0.008	34.7±5.1	105±16
75 Tyr	0.0263±0.0038	6.19±0.82	18.7±2.5	----	----	----
76 Arg	0.0168±0.005	10.9±1.4	33.2±4.4	0.037±0.010	28.1±6.6	85±20
79 Glu	0.101±0.036	10.3±1.0	31.1±3.1	0.041±0.012	34.7±9.3	105±28
80 Ser	----	----	----	0.063±0.024	15.5±2.9	46.9±8.8
81 Val	0.066±0.019	14.4±2.7	44±8	----	----	----
82 Ala	0.0128±0.0034	6.1±0.5	18.3±1.4	0.021±0.006	16.4±2.9	49.6±8.9
86 Gly	0.0077±0.0012	3.36±0.14	10.2±0.4	0.0076±0.002	5.73±0.80	17.4±2.4
87 Val	0.0101±0.0019	3.32±0.29	10±0.9	0.0198±0.0036	5.67±0.91	17.1±2.8
88 Ala	0.021±0.003	4.1±0.4	12.2±1.2	0.015±0.007	9.55±0.94	28.8±2.9
89 Tyr	0.0162±0.0024	7.8±1.2	23.4±3.7	----	----	----
96 Met	0.0336±0.0039	5.7±0.55	17.1±1.7	----	----	----
99 Ala	0.0107±0.0017	4.64±0.24	14.02±0.73	----	----	----
100 Ser	0.0092±0.0007	3.75±0.12	11.3±0.38	0.0073±0.0018	5.2±0.5	15.8±1.5
101 Phe	0.0066±0.0012	2.66±0.19	8.1±0.6	0.030±0.002	15.2±0.9	45.9±2.8

Table S5. Peak volumes of YadAM-Mx and OM signals extracted from 2D NCO spectra using Topspin 4.2.0. software.

Residue	YadAM–Mx	YadAM–OM
	Intensities (AU)	Intensities (AU)
28 Leu	----	6.40E+09
30 Thr	7.11E+09	----
31 Arg	2.50E+10	----
32 Val	1.80E+10	6.78E+09
33 Asp	3.08E+10	5.22E+09
36 Leu	4.25E+10	6.76E+09
43 Asn	----	9.46E+09
45 Leu		9.18E+09
44 Ser	4.37E+09	----
46 Phe	----	8.07E+09
49 Tyr	1.97E+10	6.70E+09
50 Gly	----	7.46E+09
52 Gly	1.42E+10	----
55 Asn	2.18E+10	----
56 Phe	1.26E+10	----
58 Ala	2.69E+10	9.08E+09
63 Tyr	1.59E+10	4.94E+09
70 Ala	4.86E+10	----
72 Gly	3.89E+10	5.37E+09
73 Ser	2.48E+10	---
74 Gly	1.87E+10	2.72E+09
75 Tyr	1.02E+10	----
76 Arg	1.27E+10	4.40E+09
79 Glu	1.40E+10	3.49E+09
80 Ser	8.55E+09	5.41E+09
81 Val	5.13E+09	----
82 Ala	2.00E+10	7.40E+09
86 Gly	4.90E+10	1.08E+10
87 Val	1.72E+10	9.52E+09
88 Ala	1.85E+10	3.71E+09
z89 Tyr	2.01E+10	----
96 Met	1.98E+10	----
99 Ala	2.77E+10	----
100 Ser	6.30E+10	1.60E+10
101 Phe	3.02E+10	6.48E+09

Table S6. MD-derived order parameters of YadAM-OM for NH vector. The dynamics are averaged from three replicas.

ResID	NH average	ResID	NH average
25 Leu	0.92	66 Ser	0.78
26 Asp	0.92	67 Gln	0.80
27 Lys	0.91	68 Ala	0.86
28 Leu	0.91	69 Leu	0.84
29 Asp	0.92	70 Ala	0.85
30 Thr	0.89	71 Ile	0.89
31 Arg	0.90	72 Gly	0.87
32 Val	0.91	73 Ser	0.89
33 Asp	0.89	74 Gly	0.82
34 Lys	0.91	75 Tyr	0.87
35 Gly	0.90	76 Arg	0.79
36 Leu	0.92	77 Val	0.77
37 Ala	0.92	78 Asn	0.71
38 Ser	0.92	79 Glu	0.52
39 Ser	0.90	80 Ser	0.50
40 Ala	0.89	81 Val	0.60
41 Ala	0.89	82 Ala	0.75
42 Leu	0.91	83 Leu	0.82
43 Asn	0.85	84 Lys	0.86
44 Ser	0.71	85 Ala	0.89
45 Leu	0.70	86 Gly	0.87
46 Phe	0.70	87 Val	0.90
47 Gln	0.65	88 Ala	0.90
48 Pro	---	89 Tyr	0.84
49 Tyr	0.62	90 Ala	0.76
50 Gly	0.49	91 Gly	0.52
51 Val	0.53	92 Ser	0.53
52 Gly	0.60	93 Ser	0.50
53 Lys	0.45	94 Asp	0.65
54 Val	0.62	95 Val	0.71
55 Asn	0.76	96 Met	0.88
56 Phe	0.80	97 Tyr	0.90
57 Thr	0.85	98 Asn	0.91
58 Ala	0.86	99 Ala	0.91
59 Gly	0.87	100 Ser	0.88
60 Val	0.87	101 Phe	0.87
61 Gly	0.87	102 Asn	0.84
62 Gly	0.86	103 Ile	0.79
63 Tyr	0.88	104 Glu	0.72
64 Arg	0.83	105 Trp	0.64
65 Ser	0.66		

Table S7. MD-derived order parameters of YadAM-OM at different time intervals.

ResID	0-110 ns	0-120 ns	0-250 ns	0-2000 ns	ResID	0-110 ns	0-120 ns	0-250 ns	0-2000 ns
25 Leu	0.92	0.92	0.92	0.91	66 Ser	0.77	0.77	0.78	0.81
26 Asp	0.91	0.91	0.91	0.91	67 Gln	0.79	0.79	0.76	0.80
27 Lys	0.92	0.91	0.91	0.91	68 Ala	0.87	0.86	0.86	0.86
28 Leu	0.92	0.91	0.91	0.91	69 Leu	0.85	0.85	0.85	0.86
29 Asp	0.92	0.92	0.92	0.91	70 Ala	0.85	0.85	0.85	0.86
30 Thr	0.90	0.90	0.90	0.89	71 Ile	0.88	0.88	0.88	0.88
31 Arg	0.89	0.89	0.88	0.90	72 Gly	0.84	0.83	0.85	0.87
32 Val	0.91	0.91	0.91	0.91	73 Ser	0.89	0.89	0.89	0.87
33 Asp	0.89	0.89	0.90	0.89	74 Gly	0.84	0.84	0.83	0.81
34 Lys	0.90	0.90	0.90	0.90	75 Tyr	0.88	0.88	0.83	0.85
35 Gly	0.90	0.90	0.90	0.90	76 Arg	0.85	0.84	0.76	0.71
36 Leu	0.92	0.92	0.92	0.92	77 Val	0.84	0.83	0.75	0.61
37 Ala	0.92	0.92	0.92	0.92	78 Asn	0.86	0.85	0.78	0.62
38 Ser	0.92	0.91	0.92	0.91	79 Glu	0.80	0.79	0.53	0.37
39 Ser	0.91	0.91	0.91	0.91	80 Ser	0.68	0.66	0.37	0.30
40 Ala	0.90	0.90	0.90	0.91	81 Val	0.74	0.75	0.45	0.46
41 Ala	0.88	0.88	0.88	0.91	82 Ala	0.87	0.86	0.70	0.59
42 Leu	0.89	0.89	0.90	0.91	83 Leu	0.88	0.88	0.85	0.71
43 Asn	0.90	0.90	0.89	0.88	84 Lys	0.89	0.88	0.88	0.85
44 Ser	0.88	0.88	0.85	0.84	85 Ala	0.88	0.87	0.87	0.88
45 Leu	0.78	0.75	0.72	0.79	86 Gly	0.87	0.88	0.88	0.88
46 Phe	0.79	0.77	0.69	0.72	87 Val	0.90	0.90	0.89	0.89
47 Gln	0.76	0.71	0.65	0.67	88 Ala	0.89	0.89	0.89	0.89
48 Pro	---	---	---	---	89 Tyr	0.83	0.84	0.82	0.83
49 Tyr	0.81	0.73	0.47	0.57	90 Ala	0.83	0.81	0.78	0.78
50 Gly	0.77	0.68	0.37	0.48	91 Gly	0.32	0.31	0.39	0.41
51 Val	0.77	0.76	0.47	0.51	92 Ser	0.59	0.56	0.57	0.55
52 Gly	0.68	0.67	0.58	0.60	93 Ser	0.53	0.53	0.53	0.57
53 Lys	0.70	0.70	0.44	0.44	94 Asp	0.70	0.68	0.70	0.73
54 Val	0.82	0.82	0.74	0.48	95 Val	0.73	0.73	0.73	0.69
55 Asn	0.87	0.87	0.82	0.69	96 Met	0.88	0.88	0.87	0.88
56 Phe	0.82	0.81	0.79	0.76	97 Tyr	0.89	0.89	0.89	0.90
57 Thr	0.86	0.84	0.81	0.81	98 Asn	0.90	0.90	0.89	0.90
58 Ala	0.84	0.83	0.80	0.83	99 Ala	0.90	0.90	0.90	0.91
59 Gly	0.85	0.85	0.84	0.86	100 Ser	0.88	0.88	0.87	0.87
60 Val	0.88	0.87	0.86	0.86	101 Phe	0.86	0.86	0.86	0.85
61 Gly	0.87	0.87	0.87	0.88	102 Asn	0.86	0.85	0.81	0.80
62 Gly	0.85	0.85	0.85	0.86	103 Ile	0.88	0.88	0.80	0.69
63 Tyr	0.88	0.88	0.88	0.88	104 Glu	0.85	0.85	0.75	0.66
64 Arg	0.88	0.87	0.86	0.85	105 Trp	0.83	0.83	0.76	0.57
65 Ser	0.63	0.63	0.55	0.63					

Table S8. Residence time of calcium ions interacting with the protein.

Replica 1		Replica 2		Replica 3	
Ion #	Res. Time (ns)	Ion #	Res. Time (ns)	Ion #	Res. Time (ns)
Ion 1	636	Ion 1	22	Ion 3	1450
Ion 2	4	Ion 8	475	Ion 6	185
Ion 4	7	Ion 9	1880	Ion 9	10
Ion 5	2	Ion 10	1	Ion 11	1
Ion 7	37	Ion 13	2	Ion 14	1
Ion 12	1	Ion 19	1	Ion 16	1
Ion 15	1	Ion 20	1	Ion 17	2
		Ion 21	1	Ion 18	2
		Ion 22	1	Ion 20	1

Table S9. Diffusion coefficient of lipids overall and near the protein.

	Replica	D × 10^{-7} (cm^2/s)	D × 10^{-7} (cm^2/s) Near Protein
Whole Membrane	1	0.51	0.52
	2	0.32	0.32
	3	0.39	0.39
PPPE	1	0.51	0.52
	2	0.33	0.32
	3	0.41	0.42
PVPG	1	0.37	0.37
	2	0.17	0.17
	3	0.23	0.23
PVCL2	1	0.53	0.53
	2	0.35	0.36
	3	0.35	0.35

References.

(1) Shahid, S. A.; Markovic, S.; Linke, D.; van Rossum, B.-J. Assignment and Secondary Structure of the YadA Membrane Protein by Solid-State MAS NMR. *Sci. Rep.* **2012**, *2*, 803.

(2) Schaefer, J.; McKay, R. A.; Stejskal, E. O. Double-Cross-Polarization NMR of Solids. *J. Magn. Reson.* **1979**, *34*, 443–447.

(3) Laage, S.; Lesage, A.; Emsley, L.; Bertini, I.; Felli, I. C.; Pierattelli, R.; Pintacuda, G. Transverse-Dephasing Optimized Homonuclear J-Decoupling in Solid-State NMR Spectroscopy of Uniformly 13C-Labeled Proteins. *J. Am. Chem. Soc.* **2009**, *131*, 10816–10817.

(4) Lamley, J. M.; Lougher, M. J.; Sass, H. J.; Rogowski, M.; Grzesiek, S.; Lewandowski, J. R. Unraveling the Complexity of Protein Backbone Dynamics with Combined 13C and 15N Solid-State NMR Relaxation Measurements. *Phys. Chem. Chem. Phys.* **2015**, *17*, 21997–22008.

(5) Stevens, T. J.; Fogh, R. H.; Boucher, W.; Higman, V. A.; Eisenmenger, F.; Bardiaux, B.; van Rossum, B.-J.; Oschkinat, H.; Laue, E. D. A Software Framework for Analysing Solid-State MAS NMR Data. *J. Biomol. NMR* **2011**, *51*, 437–447.

(6) States, D. J.; Haberkorn, R. A.; Ruben, D. J. A Two-Dimensional Nuclear Overhauser Experiment with Pure Absorption Phase in Four Quadrants. *J. Magn. Reson.* **1982**, *48* , 286–292.

(7) Öster, C.; Kosol, S.; Lewandowski, J. R. Quantifying Microsecond Exchange in Large Protein Complexes with Accelerated Relaxation Dispersion Experiments in the Solid State. *Sci. Rep.* **2019**, *9*, 11082.

(8) Lipari, G.; Szabo, A. Model-Free Approach to the Interpretation of Nuclear Magnetic Resonance Relaxation in Macromolecules. 1. Theory and Range of Validity. *J. Am. Chem. Soc.* **1982**, *104*, 4546–4559.

(9) Lipari, G.; Szabo, A. Model-Free Approach to the Interpretation of Nuclear Magnetic Resonance Relaxation in Macromolecules. 2. Analysis of Experimental Results. *J. Am. Chem. Soc.* **1982**, *104*, 4559–4570.

(10) Zhang, L.; Bouguet-Bonnet, S.; Buck, M. Combining NMR and Molecular Dynamics Studies for Insights into the Allostery of Small GTPase–Protein Interactions BT - Allostery: Methods and Protocols; Fenton, A. W., Ed.; Springer New York: New York, NY, 2012; pp 235–259.

(11) Gu, Y.; Li, D.-W.; Brüschweiler, R. NMR Order Parameter Determination from Long Molecular Dynamics Trajectories for Objective Comparison with Experiment. *J. Chem. Theory Comput.* **2014**, *10*, 2599–2607.

(12) Hicks, A.; Escobar, C. A.; Cross, T. A.; Zhou, H.-X. Sequence-Dependent Correlated Segments in the Intrinsically Disordered Region of ChiZ. *Biomolecules*. 2020, pp 1–23.

(13) Musselman, C.; Zhang, Q.; Al-Hashimi, H.; Andricioaei, I. Referencing Strategy for the Direct Comparison of Nuclear Magnetic Resonance and Molecular Dynamics Motional Parameters in RNA. *J. Phys. Chem. B* **2010**, *114*, 929–939.

(14) Hong, C.; Tieleman, D. P.; Wang, Y., Microsecond molecular dynamics simulations of lipid mixing. *Langmuir* **2014**, *30 (40)*, 11993-2001.

(15) Wang, Y.; Markwick, P. R.; de Oliveira, C. A.; McCammon, J. A., Enhanced Lipid Diffusion and Mixing in Accelerated Molecular Dynamics. *J. Chem. Theory. Comput.* **2011**, *7 (10)*, 3199-3207.

(16) Balusek, C.; Hwang, H.; Lau, C. H.; Lundquist, K.; Hazel, A.; Pavlova, A.; Lynch, D. L.; Reggio, P. H.; Wang, Y.; Gumbart, J. C., Accelerating Membrane Simulations with Hydrogen Mass Repartitioning. *J. Chem. Theory Comput.* **2019**, *15 (8)*, 4673-4686.

(17) Giraud, N.; Blackledge, M.; Goldman, M.; Böckmann, A.; Lesage, A.; Penin, F.; Emsley, L. Quantitative Analysis of Backbone Dynamics in a Crystalline Protein from Nitrogen-15 Spin−Lattice Relaxation. *J. Am. Chem. Soc.* **2005**, *127*, 18190–18201.
(18) Lewandowski, J. R.; Sein, J.; Blackledge, M.; Emsley, L. Anisotropic Collective Motion Contributes to Nuclear Spin Relaxation in Crystalline Proteins. *J. Am. Chem. Soc.* **2010**, *132*, 1246–1248.
(19) Saurel, O.; Iordanov, I.; Nars, G.; Demange, P.; Le Marchand, T.; Andreas, L. B.; Pintacuda, G.; Milon, A. Local and Global Dynamics in Klebsiella Pneumoniae Outer Membrane Protein a in Lipid Bilayers Probed at Atomic Resolution. *J. Am. Chem. Soc.* **2017**, *139*, 1590–1597.
(20) Good, D.; Pham, C.; Jagas, J.; Lewandowski, J. R.; Ladizhansky, V. Solid-State NMR Provides Evidence for Small-Amplitude Slow Domain Motions in a Multispanning Transmembrane α-Helical Protein. *J. Am. Chem. Soc.* **2017**, *139*, 9246–9258.
(21) Kurauskas, V.; Izmailov, S. A.; Rogacheva, O. N.; Hessel, A.; Ayala, I.; Woodhouse, J.; Shilova, A.; Xue, Y.; Yuwen, T.; Coquelle, N.; Colletier, J.-P.; Skrynnikov, N. R.; Schanda, P. Slow Conformational Exchange and Overall Rocking Motion in Ubiquitin Protein Crystals. *Nat. Commun.* **2017**, *8*, 145.
